# Thermo-electrochemical redox flow cycle for continuous conversion of low-grade waste heat to power

**DOI:** 10.1038/s41598-022-11817-1

**Published:** 2022-05-14

**Authors:** Jorrit Bleeker, Stijn Reichert, Joost Veerman, David A. Vermaas

**Affiliations:** 1 Department of Chemical Engineering, Delft University of Technology, 2629 HZ Delft, The Netherlands; 2 REDstack BV, Graaf Adolfstraat 35-G, 8606 BT Sneek, The Netherlands

**Keywords:** Batteries, Chemical engineering, Energy

## Abstract

Here we assess the route to convert low grade waste heat (< 100 °C) into electricity by leveraging the temperature dependency of redox potentials, similar to the Seebeck effect in semiconductor physics. We use fluid-based redox-active species, which can be easily heated and cooled using heat exchangers. By using a first principles approach, we designed a redox flow battery system with Fe(CN)_6_^3−^/Fe(CN)_6_^4−^ and I^−^/I_3_^−^ chemistry. We evaluate the continuous operation with one flow cell at high temperature and one at low temperature. We show that the most sensitive parameter, the temperature coefficient of the redox reaction, can be controlled via the redox chemistry, the reaction quotient and solvent additives, and we present the highest temperature coefficient for this RFB chemistry. A power density of 0.6 W/m^2^ and stable operation for 2 h are achieved experimentally. We predict high (close to Carnot) heat-to-power efficiencies if challenges in the heat recuperation and Ohmic resistance are overcome, and the temperature coefficient is further increased.

## Introduction

In the quest for reducing CO_2_ emissions, cutting energy losses has received major attention in the past decade. Despite various efforts to make industrial and power generating processes more efficient, 50–80% of the primary energy is dissipated as waste heat, where low-grade waste heat (up to 100 °C) forms the largest contribution^1^. Forman et al.estimated that in 2012 around 43 PWh (1.6 × 10^20^ J) of low-grade waste heat was emitted globally^1^. Although not all waste heat can be converted into power due to the conservation of entropy, the Carnot efficiency ($1-\frac{{T}_{hot}}{{T}_{cold}}$) still allows to capture 20% of the low-grade waste heat (100 °C) as power, assuming an environment of 25 °C. Converting just this fraction of the low-grade waste heat into electricity could already generate 39% of the world’s electricity consumption (22.3 PWh/year, IEA as of 2018^2^).

A major bottleneck for converting low-grade waste heat into power is the low practical efficiency of existing technologies, even compared to the Carnot efficiency. Traditionally, the organic Rankine cycle (ORC) has been used, which converts typically 4–9% of the (100–120 °C) waste heat to power^3^. The relatively low energy efficiency and the corresponding low (environmental and economic) benefits, have limited the practical application of ORC. Newer heat-to-power technologies, e.g. Reverse Electrodialysis^4–6^, Thermal Regenerable Redox-flow Batteries^7,8^ or Pressure Retarded Osmosis combined with membrane distillation^9,10^, have not shown higher energy efficiencies. Hence, a heat-to-power technology with potential for high energy efficiency is demanded.

A recent technology with high potential for efficient conversion is the Thermally Regenerative Electrochemical Cycle (TREC)^11^, which makes use of the temperature-dependent battery voltage. More energy can be obtained upon discharging at a first temperature, compared to the charging at a different temperature, generating net electrical power. Lee et al*.* has shown experimentally, using a solid Cu/Cu hexacyanoferrate (HCF) battery, that waste heat could be converted into power highly efficiently: even up to 80% of the Carnot efficiency can be reached when heat would be fully recuperated with a heat exchanger. The work by Lee et al*.* inspired the development of the TREC over the past years^12^, including a membrane free system (NiHCF, Ag/AgCl)^13^, a CoHCF based TREC (CoHFC, Ag/AgCl)^14^ and even first applications of a TREC into a combustion engine^15^ and the hot roof of a building^16^.

**Figure 1:**
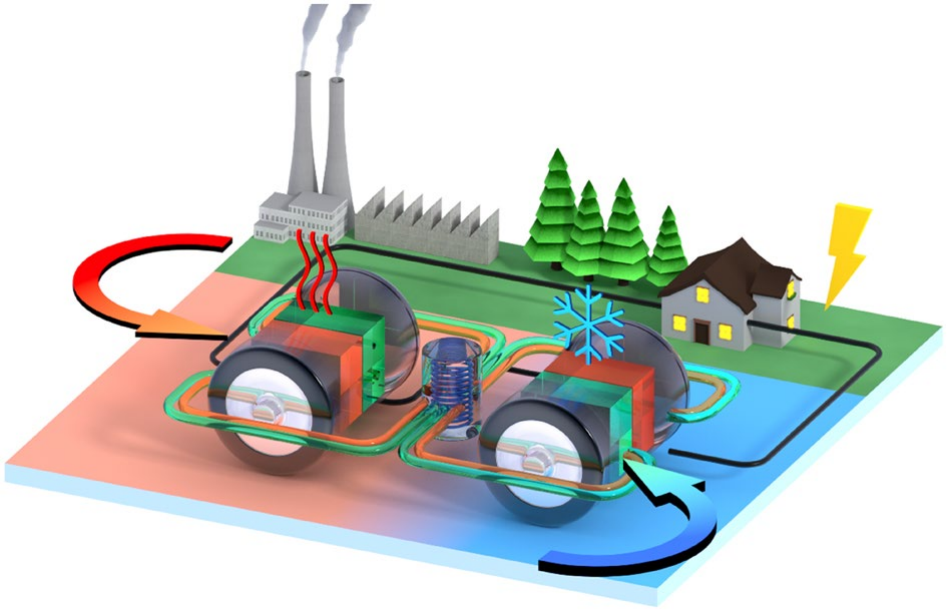
Artist impression of using two flow batteries (left battery heated; right battery cooled) to convert heat into electric power.

However, a practical drawback of the above TRECs is the slow heat transport in solids and stationary fluids. Hence, the use of a battery based on solid redox active species makes the heat recuperation unpractical, leading to long cycle times (several hours for Lee et al*.*), corresponding to a low power density (1.2 mW/g)^11^. Redox Flow Batteries (RFBs) could leverage the intrinsic facile heating and cooling of liquid redox active species in heat exchangers, which makes them attractive candidates for the TREC. Using two redox flow batteries, one operating at low temperature and one operating at high temperature, could create a redox flow cycle for continuous heat-to-power conversion (Fig. 1). Several electrolytes have been suggested, such as the All-Vanadium RFB by Reynard et al.^17^ and a V^2+^/V^3+^, Fe(CN)_6_^3−^/Fe(CN)_6_^4−^ battery by Poletayev et al.^18^. However, the All-Vanadium RFB suffers from chlorine formation and V_2_O_5_ precipitation at elevated (> 60 °C) temperatures, and the electrolytes selected by Poletayev et al*.* will be difficult to separate with a monopolar ion membrane.

Here we present a RFB that has high potential for continuous heat to power conversion. Our RFB is designed based on hexacyanoferrate and iodide/polyiodide redox couples:

- I_3_^−^ + 2e^−^ ⟷ 3 I^−^ E^0^ = 0.54 V vs SHE
- Fe(CN)_6_^4−^ ⟷ Fe(CN)_6_^3−^ + e^−^ E^0^ = 0.36 V vs SHE

We evaluate the potential of this route via the criteria for thermo-electrochemical RFBs, and present a system for continuous heat to power conversion with a TREC based on RFBs. The same chemistry has been reported by Qian et al*.* recently^19^, and has shown promise for heat to power recovery. In this work, we report a higher temperature coefficient and power density, by changing the concentrations of the electrolytes and using a KCl supporting electrolyte.

## Working principle

### The temperature coefficient of redox reactions:

The concept of the thermo-electrochemical flow cell relies on the dependency of the redox potential on temperature, similar to the Seebeck effect in semiconductor physics. This temperature dependency of redox reactions is sometimes mistakenly accredited to the Seebeck effect with corresponding Seebeck coefficient, which is the actually the charge drift driven by a temperature gradient^20^. This temperature dependency is dominantly due to changes in entropy between reduced and oxidized species (see SI Note 1). The change in redox potential is described as:

1 $${E}_{i}={{E}_{i}}^{0}+{\alpha }_{i}\left(T-{T}^{0}\right)$$

2 $${\alpha }_{i}\approx \frac{\Delta {S}_{R}}{nF}$$

In which ${E}_{i}$ is the redox potential (in V) of redox reaction *i*, ${E}^{0}$ is the standard potential at 298 K (in V), $\alpha$ is the temperature coefficient (in V/K), $T$ is the reaction temperature (in K), ${T}^{0}$ is the standard temperature (298 K), $\Delta {S}_{R}$ is the reaction entropy for a reduction reaction, $n$ is the number of electrons involved and $F$ is the Faraday constant (96,485 C/mol). The concentration dependency of the temperature coefficient can be described as follows^21^:

3 $$\alpha = {\alpha }_{0}-\frac{R}{nF}\mathrm{ln}(Q)$$

Here α_0_ is the temperature coefficient at standard concentrations and Q is the reaction quotient of the redox reaction (see SI Note 1 for a derivation and comparison of temperature coefficients at different concentrations).

### Thermo-electrochemical energy

The battery’s open circuit voltage, OCV (in V), arises from the potential difference between the redox potentials of species 1 and 2, where Q is the reaction quotient of the cell reaction:

4 $$OCV={{E}_{1}}^{0}-{{E}_{2}}^{0}+\left({\alpha }_{1}-{\alpha }_{2}\right)\left(T-{T}^{0}\right)-\frac{RT}{nF}\mathrm{ln}(Q)$$

The change in cell voltage at different temperatures can be leveraged when using two batteries: one battery in which both anolyte and catholyte operate at a high temperature, and a second battery in which both reactions occur at a low temperature. The difference in OCV between the hot battery and the cold battery is:

$$OC{V}_{hot}-OC{V}_{cold}=\left({\alpha }_{1}-{\alpha }_{2}\right)\left({T}_{hot}-{T}_{cold}\right)-\frac{R{T}_{hot}}{nF}\mathrm{ln}\left({Q}_{hot}\right)+\frac{R{T}_{cold}}{nF}\mathrm{ln}\left({Q}_{cold}\right)$$

5 $$={\alpha }_{cell}\Delta T-\frac{R}{nF}\mathrm{ln}\left(\frac{{{Q}_{hot}}^{{T}_{hot}}}{{{Q}_{cold}}^{{T}_{cold}}}\right)$$

**Figure 2:**
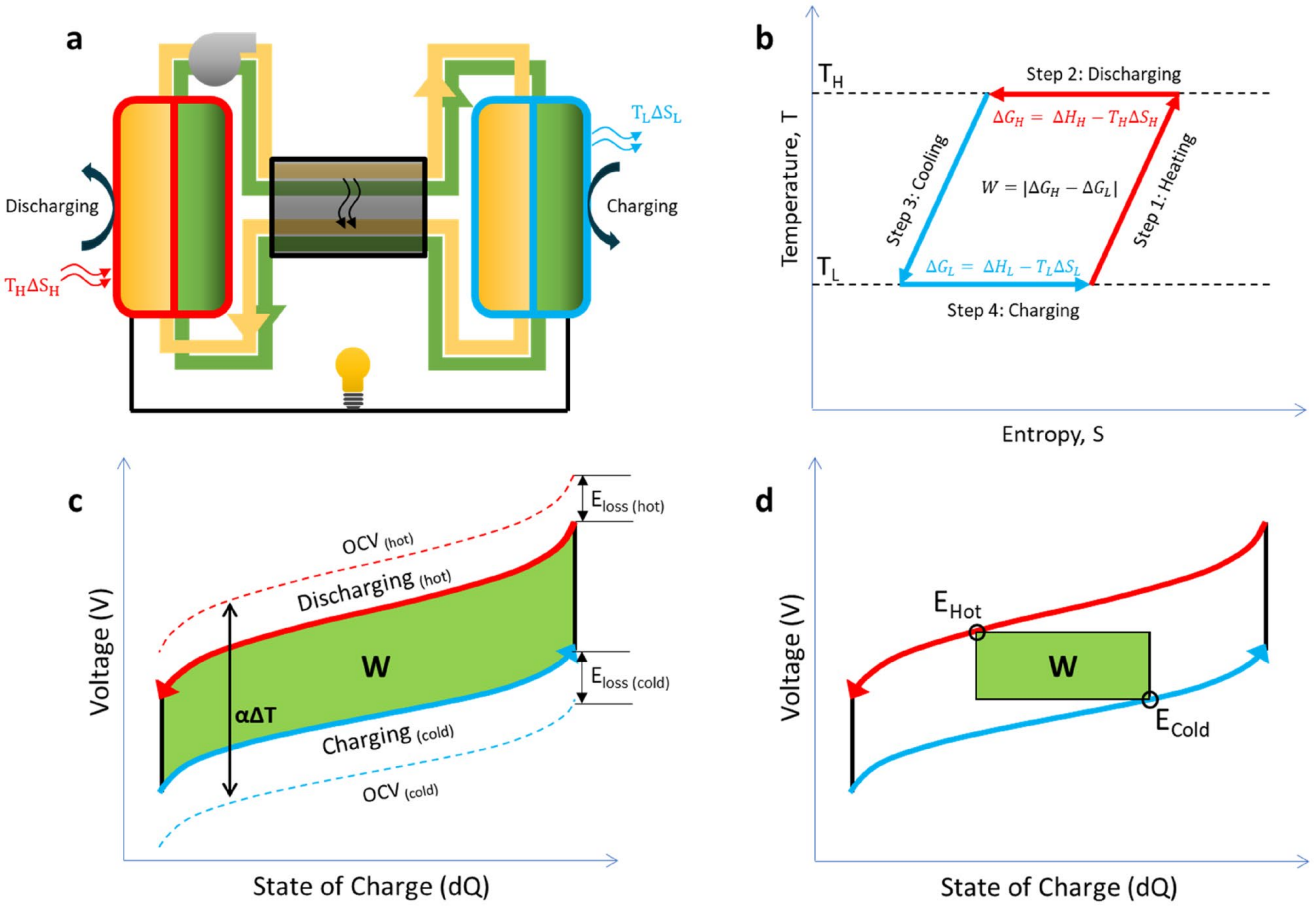
Concept of thermally regenerative redox flow cycle and thermodynamic diagrams. (**a**) The electrolyte circulation between a hot RFB (red, discharging) and a cold RFB (blue, charging at lower voltage), with a heat exchanger in the center. (**b**) Corresponding ST-diagram of the charging and discharging cycle. (**c**) Battery voltage as a function of State of Charge, for hot and cold state, indicating the maximum obtainable work (W), due to the change in cell voltage at different temperatures (αΔT) and including overpotential losses (E_loss_). (**d**) Battery voltage as a function of State of Charge, including the obtainable work when charging and discharging at a single voltage (i.e., continuous mode).

In which Q_hot_ and Q_cold_ indicate the reaction quotient at the operating conditions of the hot and cold battery respectively and α_cell_ is the temperature coefficient of the combined electrolytes. The difference in OCV drives an electric current between the hot and the cold battery, which can be used as a power source (Fig. 2a). The maximum power that can be extracted from the difference in battery voltage is given by the Kirchhoff law (Eq. 6), which assumes a constant battery resistance, *R* (in $\Omega {\mathrm{ m}}^{2}$) and a constant cell voltage. The maximum power density *P*_*max*_ (in W/m^2^) is then given by:

6 $${P}_{max}=\frac{{\left({\alpha }_{cell}\Delta T-\frac{R}{nF}\mathrm{ln}\left(\frac{{{Q}_{hot}}^{{T}_{hot}}}{{{Q}_{cold}}^{{T}_{cold}}} \right)\right)}^{2}}{4({R}_{hot}+{R}_{cold})}$$

### Thermodynamic analysis

From a thermodynamic point of view, the battery process can be illustrated in a TS-diagram (Fig. 2b). When the heat from the hot RFB outflow is recuperated via a heat exchanger and no losses are included, the Carnot efficiency can be obtained (see SI Note 2).

The expected voltages at the hot and cold RFB are obtained from Eq. (4). When including electrical losses (Ohmic resistances, kinetic overpotentials) a V-dQ diagram is established (Fig. 2c,d). The maximum work is obtained when the battery voltage is continuously adapted to the individual battery potentials (Fig. 2c). This resembles a batch mode operation, or a segmentation of electrodes that can be individually controlled (Fig. S-1). A more practical operation is a continuous, single-stage, battery mode. However, this single charge and discharge voltage, E_hot_ and E_cold_, respectively, compromises the obtainable work (Fig. 2d).

## Methods

### Materials and electrolyte preparation

All chemicals were purchased from commercial suppliers (Alfa Aesar, KGaA, VWR International), and were at least of 99% purity and were used without further purification. Demineralized water was used to prepare the electrolytes.

The temperature coefficient of the individual hexacyanoferrate redox couple was measured in 0.1 M K_3_Fe(CN)_6_, 0.1 M K_4_Fe(CN)_6_·3H_2_O and 0.3 M KCl. The temperature coefficient of the individual polyiodide redox couple was measured in 0.01 M KI, 0.001 M I_2_, 1 M KCl. KCl was added to raise the K^+^ concentration to ~ 1 M.

The measurement of the cell temperature coefficient and the proof of concept were done at higher concentrations. The hexacyanoferrate electrolyte consisted of 0.3 M K_3_Fe(CN)_6_, 0.3 M K_4_Fe(CN)_6_·3H_2_O and 0.35 M KCl. KCl was added in order to obtain 2 M fully dissociated K^+^ ions. The polyiodide electrolyte consisted of 0.9 M KI, 0.3 M I_2_ and 1.1 M KCl. The solution was stirred overnight to dissolve all I_2_.

### Individual temperature coefficient measurements

The temperature coefficients were measured by performing cyclic voltammetry at various temperatures between 20 and 55 °C in 50 mL of electrolyte on a hotplate stirrer (IKA C-MAG HS7) with two Pt wires as working and counter electrodes and a Ag/AgCl reference electrode (ProSense, double junction). We used a custom-made 30 cm long glass salt bridge filled with 1 M KCl (Fig. S-2b) to ensure the Ag/AgCl reference electrode did not heat up.

The cyclic voltammetry was performed using a potentiostat (Ivium CompactStat.h10800) with a scan rate of 50 mV/s, cycling between − 0.2 and 0.5 V vs Ag/AgCl @20 °C for hexacyanoferrate and 0.2 and 0.6 V vs Ag/AgCl @20 °C for polyiodide. The halfway potential (E^1/2^) was calculated by taking the average of the cathodic and anodic peak positions^22^, which were measured against a Ag/AgCl reference electrode (ProSense B.V.) at 20 °C. The temperature coefficient was taken as the slope of the linear fit of E^1/2^ versus T data.

### Single flow cell characterization

The cell temperature coefficient (i.e., combined with both redox couples) was experimentally assessed using a custom-made PTFE flow cell with graphite sheet electrodes with a geometrical surface area of 86.6 cm^2^ and FKM gaskets (see Fig. S-4a). The hexacyanoferrate and polyiodide flow compartments were separated with a Selemion CMV cation exchange membrane. A flow diagram with the various components can be seen in Fig. S-11. We used 0.5 L of both electrolytes, of which ~ 0.25 L was always in the system. The temperature coefficient was calculated by measuring the open cell voltage (OCV) at various temperatures between 20 and 40 °C and performing a linear fit through the data. The OCV was measured with a potentiostat (IviumStat.h standard). The electrolyte was heated by coiling up part of the tubing and submerging it in a heating bath (Julabo Corio C-B19). The electrolyte temperature was measured at the inlet and outlet of the flow cell with four K-type thermocouples (TC Direct-405-011) and read out with a NI 9213 module. The OCV was measured once the temperature was stable (± 0.5 °C for 5 min). The temperature of the heating bath was then raised for the next measurement. The electrolyte was pumped (Masterflex Precision peristaltic pump EW-07528–10) through PTFE tubing, which was insulated with PE foam. The flow cell was not thermally insulated. The resistance of a single flow cell was measured with chronopotentiometry, in a range of currents for 60 s each (10.3 to − 10.3 A/m^2^, with increments of 1.15 A/m^2^). The resistance was calculated by a linear fit through the measured voltages.

### Proof of concept measurements

A flow diagram of the entire setup can be seen in Fig. S-12. The temperature of the flow cells is controlled with a cooling bath (Julabo Corio CD-601F), heating bath (Julabo Corio C-B19) and a glass heat exchanger (custom made by Squall Instruments, 1 m long, 70 double coils, operating in counter-flow Fig. S-5). The temperature was measured at the inlet and outlet of both flow cells. The operating temperature of the flow cell was assumed to be the mean outlet temperature of the two electrolytes.

**Figure 3:**
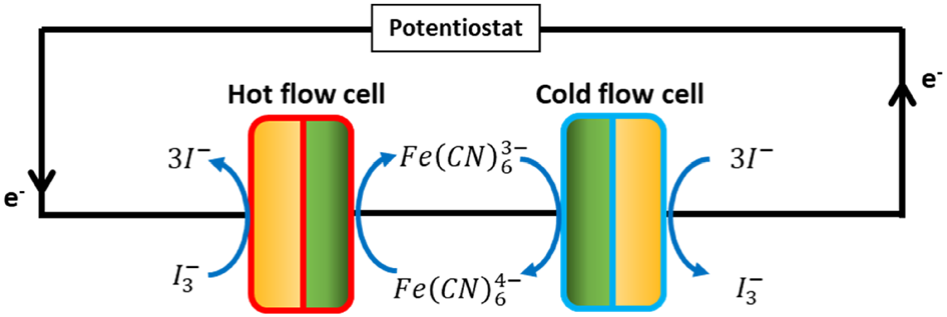
Schematic representation of the electrical connections during the proof of concept measurements. The hot flow cell is discharging (spontaneous reaction), the cold cell is charging (electrolysis).

The power output of the system was experimentally validated with two flow cells operating at different temperatures. An electrical loop was made between the two cells and the potentiostat (IviumStat.h standard), see Fig. 3. A chronopotentiometry method was run at 13 different current densities with increments of 1.73 A/m^2^ for 60 s each. The power density was calculated by multiplying the current density with the obtained voltage difference over the two cells (E_Cell hot_ − E_Cell cold_). Note that the area in power and current density are for a single flow cell. The system was allowed to run for 3 h at the maximum power output to test the stability.

### Modelling

The heat to power efficiency calculations were performed in a Python script. The used equations and assumptions are stated in SI Note 6.

## Results

### Selection of suitable redox couples

To perform power generation via a thermo-electrochemical RFB in practical heat-to-power applications, a RFB system should meet the following requirements:

1. The RFB needs to consist of two solute redox systems with a large difference in temperature coefficients to maximize α_cell_ (= α_1_ − α_2_).
2. The redox species need to have a high solubility (allowing small water volume heating/cooling), fast kinetics (low overpotentials) and stability over a temperature range of at least 10–80 °C (allowing for high ΔT)
3. All redox active species need to have the same valence sign, to allow separation with a (monopolar) ion-exchange membrane. A bipolar membrane could be used alternatively, but the current state-of-the-art bipolar membranes would result in unacceptable large energy losses^23^.

The most mature RFB, the all-vanadium RFB, unfortunately does not meet criteria 1 and 2. Both the V(II)/(III) and the V(IV)/V(V) couple have positive temperature coefficients^24^ and the V(V)-ions can irreversibly precipitate above 40 °C^25^. This could be solved by with a mixed acid electrolytes, however that can result in Cl_2_ gas formation at 60 °C^17^. Also Br_2_-based batteries are unsuitable, due to the high vapour pressure of bromine at elevated temperatures (boiling point is 59 °C at atmospheric pressure).

Other examples of RFB based systems targeting heat to power is consisted Cu(NH_3_)_4_^2+^/Cu(NH_3_)_2_^+^ or V^2+^/V^3+^ and Fe(CN)_6_^3−^/Fe(CN)_6_^4−^. These electrolytes have the highest reported temperature coefficients for TRECs to date (− 2.9 mV/K and − 3.0 mV/K respectively)^18,26^, but do not meet the 3rd criterion. Hence, despite the predicted high efficiencies, the first system suffered from ion crossover causing precipitation of Cu_2_Fe(CN)_6_ and a high internal resistance. The V^2+^/V^3+^, Fe(CN)_6_^3−^/Fe(CN)_6_^4−^ was not tested for stability, but will likely suffer from vanadium crossover as the electrolytes are only separated by a Nafion cation exchange membrane.

**Figure 4:**
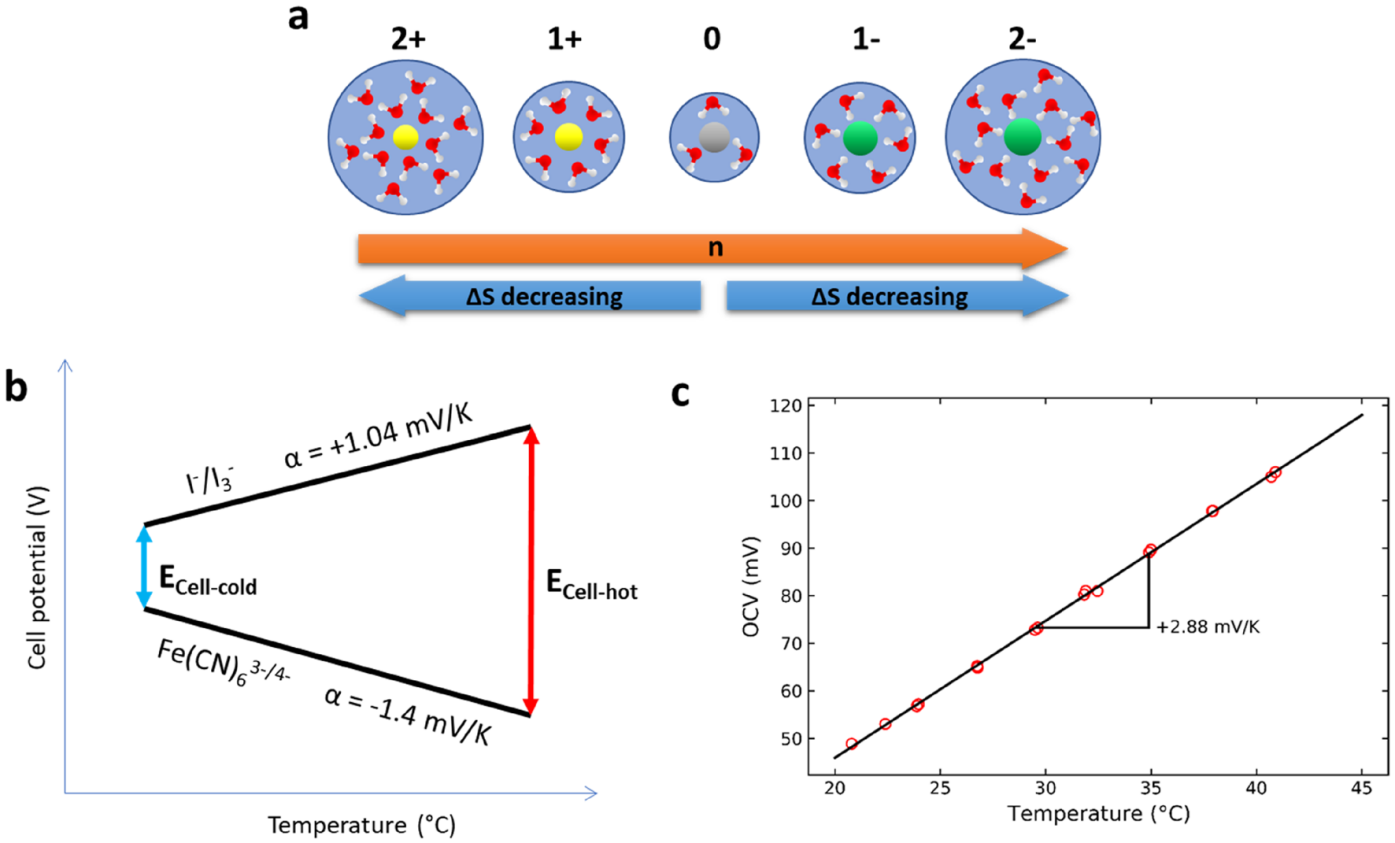
(**a**) The suggested change in entropy upon valence change of an ion. (**b**) Redox potentials as a function of temperature for individual redox reactions, obtained via cyclic voltammetry between 20 and 55 °C (Fig. S-2a). To avoid temperature changes in the Ag/AgCl reference electrode, a long glass salt bridge was used (Fig. S-2b), (**c**) and for the assembled RFB cell, at approximately 50% State of Charge (SOC). The slope represents the temperature coefficients.

The sign of the temperature coefficient appears to be correlated with the sign of the valence of the redox active species that undergo a simple one-electron transfer reaction. For example redox couples consisting of cations, e.g. Fe^3+^/Fe^2+^, Cu^2+^/Cu^+^ and Co^3+^/Co^2+^, all have positive temperature coefficients^21,27^, while their anion counterparts, e.g. Fe(CN)^3−^/Fe(CN)^4−^ and MnO_4_^−^/MnO_4_^2−^, have negative coefficients^11,27^ (see SI Note 3 for 54 examples from literature sources). We hypothesize that the change in entropy (and thus the temperature coefficient) is dominated by the size of the ion hydration shell, which grows upon increased valence magnitude (Fig. 4a). Unfortunately, this property makes it difficult to satisfy both criterion 1 and 3.

To match all three criteria we chose the polyiodide redox couple, as it is not a simple one-electron transfer reaction and deviates from the rule. We ended up a flow cell with I^−^/I_3_^−^ and Fe(CN)_6_^3−/4−^ chemistry. The reactions below are written in the discharging (galvanic) form below:

- I_3_^−^ + 2e^−^ → 3I^−^
- Fe(CN)_6_^4−^ → Fe(CN)_6_^3−^ + e^−^

The temperature coefficients of I^−^/I_3_^−^ and Fe(CN)_6_^3−/4−^ were experimentally determined as + 1.04 mV/K and − 1.40 mV/K, respectively (Fig. S-2a). The values agree with reported values in literature, as the temperature coefficient of Fe(CN)_6_^3−/4−^ is well documented to be around − 1.4 mV/K^28,29^ and I^−^/I_3_^−^ agrees with tabulated values when corrected for the concentrations we use here (see SI Note 1). The combination of the two electrolytes predicts a cell-temperature coefficient of + 2.44 mV/K (Fig. 4b).

K_3_Fe(CN)_6_ and K_4_Fe(CN)_6_ are both soluble up to 0.4 M and have been used in literature at 80 °C, remaining stable for over 90 days of operation^28,30^. The polyiodide couple is reported in RFBs well above 1 M^31^ and has a stable cycling performance^32,33^. Also, triiodide electrolytes have been reported well over 80 °C^34^ and we therefore assumed the redox couple is stable over a large temperature range.

As all active species in the selected redox couples are anions, they are separable by a cation exchange membrane (CEM). The triiodide equilibrium, I_3_^−^ ↔ I_2_ + I^−^, is strongly balanced towards I_3_^−^, which minimizes the potential crossover of I_2_. Also, Ding et al. have shown that a Nafion membrane could be used to separate these two electrolytes for 500 cycles with negligible cross-over effects^33^. Moreover, the combination of these redox couples results in a low cell potential of 0.18 V (at room temperature). While this low OCV disfavours to use this battery chemistry for energy storage, it benefits the use for heat-to-power as it minimizes self-discharge of the electrolytes.

### Single flow cell characterization

The OCV of a flow cell exhibits a linear dependence on temperature between 20 and 40 °C (Fig. 4c), indicating a constant α_cell_. The obtained temperature coefficient is + 2.88 mV/K, slightly larger than the individual coefficients that were measured through cyclic voltammetry (+ 2.44 mV/K). This is likely due to the different ratio of KI to I_2_ in the electrolyte in the flow cell experiments, causing a different Q in Eq. (3) and explaining a change in temperature coefficient. The temperature coefficient is possibly affected by the change in reaction entropy to other polyiodides (e.g. I_5_^−^ or I_7_^−^) that form at higher iodine concentrations^34^ or by the change of reaction towards I_2_ instead of I_3_^−^, which has a higher temperature coefficient^27^.

The area resistance of a single flow cell is 7.1 Ω cm^2^ at 22 °C and decreases to 3.6 Ω cm^2^ at 40 °C. We assume the resistance follows^35^:

7 $$R\left(T\right)=\frac{{R}_{0}}{1+\theta (T-{T}_{0})}$$

Here R_0_ (7.1 Ω cm^2^) is the resistance at reference temperature T_0_ (22 °C) and θ is a fitting parameter (0.060 K^−1^ for this case, see Fig S-3).

### Comparison with other reported systems

We can compare our I^−^/I_3_^−^–Fe(CN)_6_^3−/4−^ system to other thermo-electrochemical systems, by adopting their dimensionless Figure of Merit (Y). Y is the ratio of available electrical energy to the required absorbed heat; a higher figure of merit allows one to get a higher heat to power efficiency at a fixed heat exchanger efficiency. Lee et al.^11^ defined Y as:

8 $$Y=\frac{\left|\alpha \right|{q}_{c}}{{c}_{P}}$$

Here |α| is the absolute temperature coefficient of the system, q_c_ is the specific charge capacity and c_P_ the specific heat capacity of the electrodes and electrolyte. Ohmic and Nernstian losses are ignored for all systems (see SI Note 4 for more details). Even though other reported TREC systems use solid redox species and higher concentrated electrolytes, the system we report here has a comparable figure of merit of 0.021 (Table 1), while still having the benefits of liquid handling. A lower Y is expected for all-liquid based systems, due to the relatively high heat capacity of water, and poses additional requirements for the heat recuperation. However, the concept of redox flow batteries, allowing liquid–liquid heat exchangers, easily improves the heat transfer flux by an order of magnitude compared to stationary with solid redox species, which justifies the 2–3 fold lower Y for practical TREC systems.

**Table 1:** Comparison with other reported low grade heat TREC systems.

Electrolytes	|α_Cell_| (mV/K)	Figure of merit—Y	System architecture	References
Fe(CN)_6_^3−/4−^, V^3+^/V^2+^	3.00	0.032	Liquid flow cell	^18^
VO_2_^+^/VO^2+^, V^3+^/V^2+^	1.16	0.013	Liquid flow cell	^17^
Fe(CN)_6_^3−/4−^, Cu(NH_3_)_4_^2+^/Cu(NH_3_)_2_^+^	2.9	0.033	Liquid stationary cell^**a**^	^26,36^
CuHCF, Cu/Cu^2+^	1.20	0.068	Solid + electrolyte	^11,36^
Fe(CN)_6_^3−/4−^, FeHCF	1.45	0.059	Solid + electrolyte	^36,37^
NiHCF, Ag/AgCl	0.74	0.034	Solid + supporting electrolyte	^13,36^
Fe(CN)_6_^3−/4−^, I^−^/I_3_^−^	1.9	0.016	Liquid flow cell	^19^
Fe(CN)_6_^3−/4−^, I^−^/I_3_^−^	2.88	0.021	Liquid flow cell^b^	This work

The figure of merit Y for our work is based on using full range in SOC; smaller ΔSOC may be still relevant for a continuous operation with a single electrode pair per battery (see Fig. 2D), and will result in a smaller figure of merit.

^a^For 0.5 M redox active species. Deposition of Cu_2_Fe(CN)_6_ at the BaSO_4_ precipitate membrane prevented continuous operation.

^b^For 0.3 M redox active species. The maximum solubility allows concentrations up to 0.5 M, which would yield Y = 0.033 if the temperature coefficient remains the same for this concentration change.

### Proof of concept

**Figure 5:**
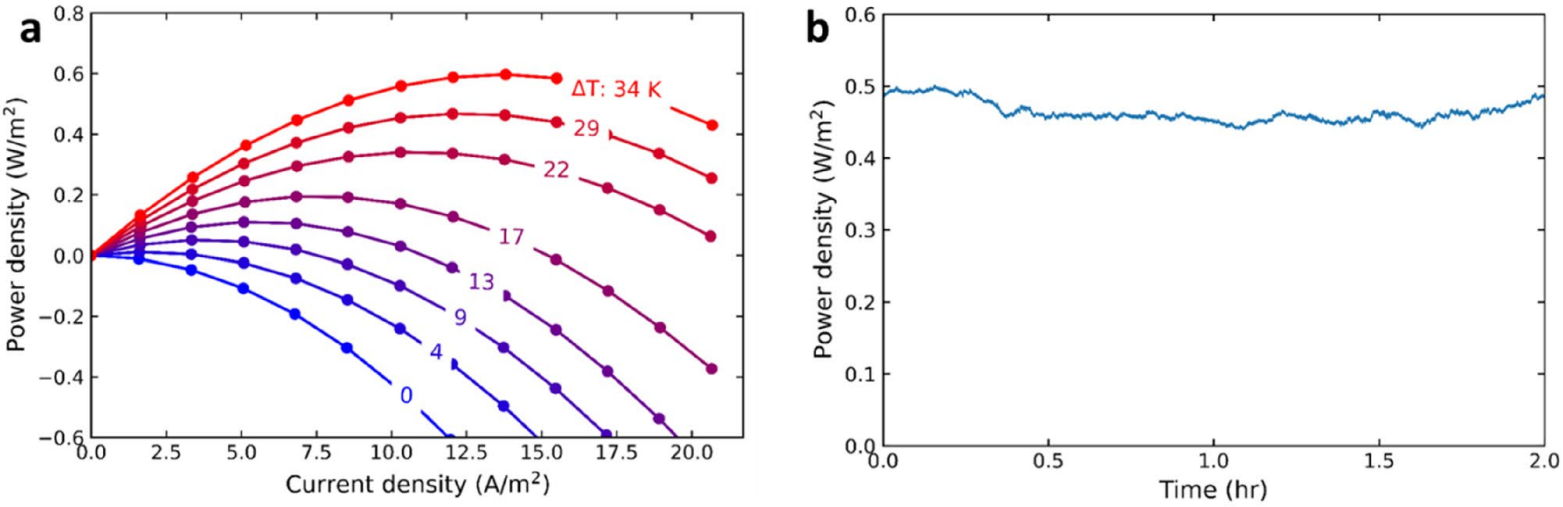
(**a**) Experimental power density as a function of applied current density, for a series of temperature difference (ΔT) between the hot battery and cold battery. The cold battery temperature is 20–22 °C for all experiments. (**b**) Power density of the system over 2 h, while operating at a ΔT of 34 K and 13.4 A/m^2^. The area (m^2^) in these figures is the electrode area of a single flow cell.

The heat-to-power performance of the polyiodide/ferrocyanide RFB was evaluated in a continuous flow setup with a cold charging and hot discharging flow cell connected in a loop as per Fig. 2a (more detailed in Fig. S-4b). Figure 5a shows the power density versus current densities for various temperature differences between the hot and cold cell. We achieved a maximum power density of 0.6 W/m^2^ at 13.8 A/m^2^ and a temperature difference of 34 °C. At this current density, the hot and cold cell are cycling between a state of charge of 50.0% and 51.2%. The maximum power density shifts to higher current densities at higher temperature intervals because both the driving force is larger (larger difference OCV_hot_ – OCV_cold_) and the Ohmic resistance is lower at higher temperatures. Still, the optimum current density is relatively small compared to commercialized RFBs, limiting also the power densities, due to the high (non-optimized) Ohmic resistance of the system (see Fig. S-3).

At the maximum power density in Fig. 5a, 50% of the available energy is converted into electricity. Here 40% of the energy is lost in Ohmic losses and activation overpotential and 10% as concentration overpotential. With perfect heat recovery, the present, non-optimized flow cells would obtain a heat to power efficiency of 5.2% (see SI Note 5 for the derivation). In the present setup, however, with limited glass heat exchangers (Fig. S-5), and poor insulation of the flow cells and tubing, an overall heat-to-power efficiency of 0.004% was obtained.

The power density of the system, evaluated for 2 h (Fig. 5b), remained relatively stable around 0.46 W/m^2^. The fluctuations in the figure are due to small temperature changes and minor changes in concentration as the electrolyte vessels were not continuously stirred.

## Outlook

Given the early stage of development and the modest power density/energy efficiency, substantial engineering improvements are necessary to make a RFB based thermo-electrochemical cell feasible for practical operation. The temperature coefficient reduced by 0.21 mV/K after 20 h of cycling (Fig. S-6), likely due to I_2_ migration across the membrane. We also observed corrosion by iodine and deposition of Prussian blue on the electrodes and membrane (Fig. S-7). Both the membrane and electrodes were not selected for long-term stability in this chemistry. Also, our current experimental design is limited by the high internal resistance and poor insulation.

**Figure 6:**
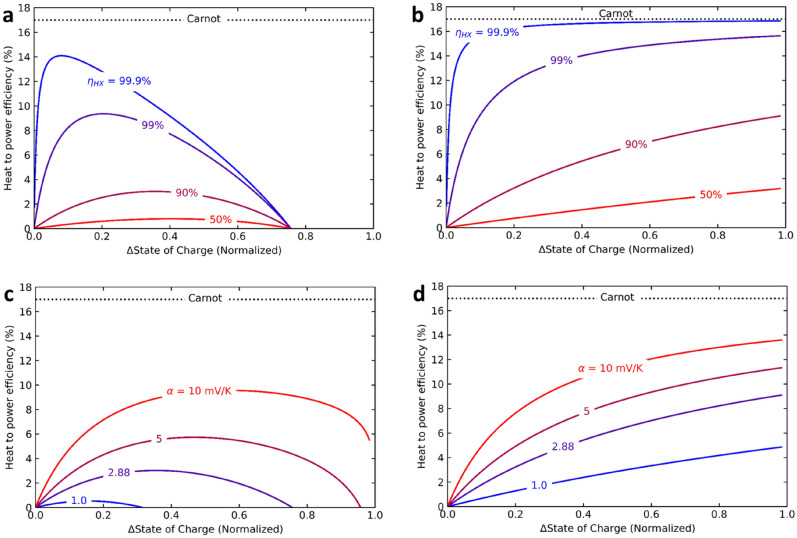
Simulated heat to power efficiency, as a function of difference in SOC, in a continuous mode (panel **a**) and batch mode (panel **b**), with hypothetical efficiencies of a heat exchanger. Results in panel a and b are obtained using T_hot_ = 80 °C, T_cold_ = 20 °C, concentrations for Fe(CN)_6_^3−^, Fe(CN)_6_^4−^ and I_2_ 0.3 M, that of I^−^ = 0.9 M, and α =  + 2.88 mV/K. Results in panel (**c**,**d**) are simulated for different temperature coefficients at a heat exchanger efficiency of 90%. Ohmic losses are ignored in these calculations.

To assess the potential of the reported system we calculated the heat-to-power efficiency of the system while varying the ΔSOC, heat exchanger efficiency, temperature coefficient, heat capacity, concentration and Ohmic losses for an optimized system (see SI Note 6). Figure 6a shows the simulated heat-to-power efficiency vs ΔSOC for various heat exchanger efficiencies for a RFB system in continuous mode. Relying on heat recuperation only, without further improving the temperature coefficient or cell operation, will be insufficient to reach substantially high energy efficiency in continuous flow mode. Even at very high heat exchanger efficiencies (99.9%) a large fraction of energy is lost and only a maximum heat to power efficiency of 14% can be obtained. At a more realistic heat exchanger efficiency of 90%, only 3% of the waste heat is recovered as electrical power. The maximum heat-to-power efficiency shifts to higher ΔSOC for lower heat exchanger efficiencies, to reduce the amount of fluid that needs to be heated/cooled in poorer heat exchangers. At a ΔSOC of 0.75, the difference in hot/cold cell voltages becomes 0, due to the hysteresis in the V-dQ curve (Fig. 2d).

Figure 6b shows the heat-to-power efficiency for a system in batch mode. In batch mode the complete area of the V-dQ curve can be harvested (Fig. 2c), and hence higher efficiencies can be achieved. At a ΔSOC of 1 and a perfect heat exchanger the system will approach the Carnot efficiency. Even with a more realistic heat exchanger efficiency of 90% and ΔSOC = 0.5, more than 6% of the heat can be converted into electricity in batch mode, bettering the current state-of-the-art heat-to-power technologies. Figure 6c,d show the effect of the temperature coefficient on the heat-to-power efficiency for a RFB in continuous and batch mode, respectively. As the larger temperature coefficient increases the vertical shift of the V-dQ curves, the point of zero work also shifts to higher ΔSOC. The temperature coefficient of our system could be increased in practice by the addition a volume fraction of an organic solvent. The temperature coefficient ferro/ferricyanide redox couple has been shown to amplify up to – 4.2 mV K^−1^^28,29^ with additives. Preliminary experiments have shown that the addition of ethanol to the triiodide electrolyte results in a more positive temperature coefficient, resulting in a very large cell temperature coefficient (Fig. S-10). The addition of organic solvents does however reduce conductivity and increase Ohmic losses. Other ways to increase the temperature coefficient could be to design a system around polysulfide (− 4.08 to − 5.33 mV K^−1^)^38^ or a redox reaction with a large ΔS due to a phase transition^39,40^.

Finally, the effect of the concentration, heat capacity and Ohmic losses on the heat-to-power efficiency is assessed (Figs. S-8, S-9). Provided that the heat capacity and maximum concentration have intrinsic limits, the Ohmic resistance is the only remaining knob for optimizing the heat-to-power efficiency. A 50 mV Ohmic loss (over the entire two cell circuit) almost halves the heat-to-power efficiency (at current α = 2.88 mV/K, η_HX_ = 90%, 0.3 M active species). Improvements on the cell design, such as a zero-gap flow cell, together with a low resistive membrane could be used to minimize the Ohmic resistance and allow for much higher current densities^41^. Implementation of high surface area electrodes could improve the kinetics, further reducing electric losses in the system.

Hence, this proof of concept of a RFB-based system for continuous heat-to-power conversion should gain improvement in the realm of higher temperature coefficient and low Ohmic resistances to fully unlock its potential for effective conversion of waste heat to power.

## Supplementary Information

Supplementary Information.

## Data Availability

The data supporting the findings of this study are contained within the paper and its associated Supplementary Information. All other relevant data is available from the corresponding author upon reasonable request and in the Zenodo repository at https://zenodo.org/record/6337819.
