# Supplementary material for: Thermo-electrochemical redox flow cycle for continuous conversion of low-grade waste heat to power
Source: Sci Rep. 2022 May 14;12:7993. doi: 10.1038/s41598-022-11817-1 (PMC9107487; doi:10.1038/s41598-022-11817-1)
Supplement: Supplementary file 1 — Supplementary Information. [file 41598_2022_11817_MOESM1_ESM.pdf]

Supplementary Information

Corresponding to

Thermo-electrochemical redox flow cycle for continuous conversion of low-grade waste heat to power

Jorrit Bleeker [1], Stijn Reichert [1], Joost Veerman [2], David A. Vermaas [1,*]

[1]Department of Chemical Engineering, Delft University of Technology, 2629 HZ Delft, The Netherlands

[2]REDstack BV, Graaf Adolfstraat 35-G, 8606 BT Sneek, The Netherlands

email: d.a.vermaas@tudelft.nl

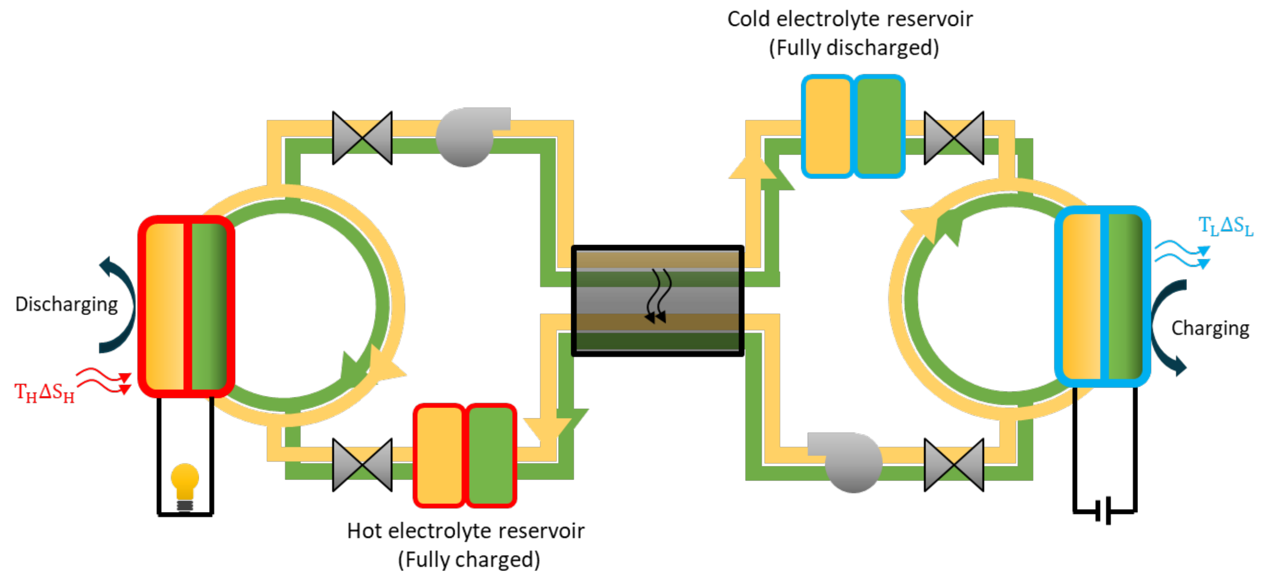

Figure S-1. *Concept of thermally regenerative redox flow cycle operating in a batch mode. The figure illustrates the electrolyte circulation between a hot RFB (red, discharging) and a cold RFB (blue, charging at lower voltage), with a heat exchanger in the center. The electrolyte is circulated until it is fully discharged/charged before it will be pumped to the other reservoir of the other flow cell.*

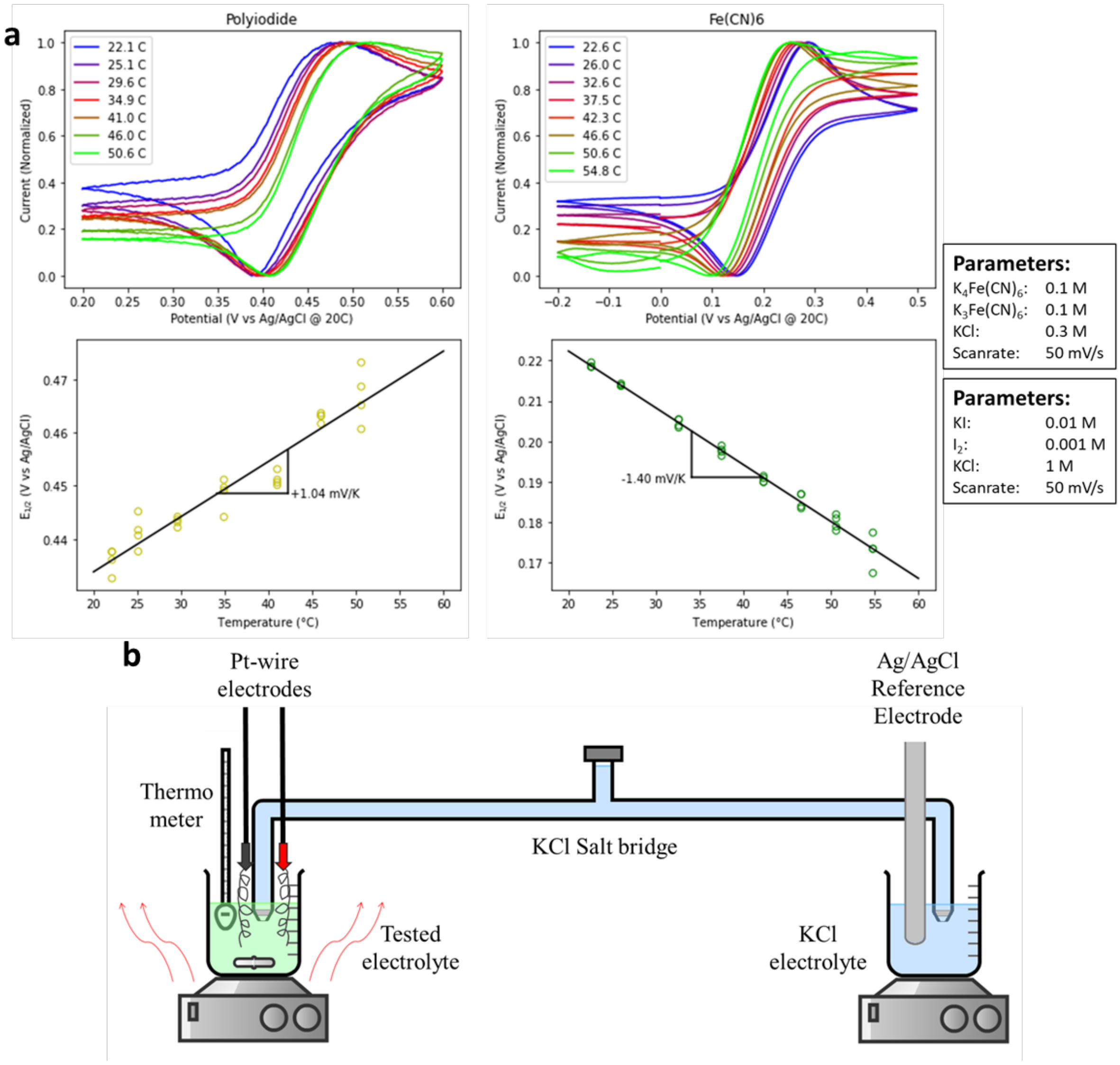

Figure S-2. a. *Top panels: CV scans of the two electrolyte couples at different temperatures. Bottom panels: Half-cell potentials (from the CV scans) plotted against the temperature. The slope of the linear fit is the temperature coefficient.* ***b.*** *Schematic representation of the temperature coefficient measurement setup with a long salt bridge. The reference electrode is kept at room temperature, while the tested electrolyte is heated.*

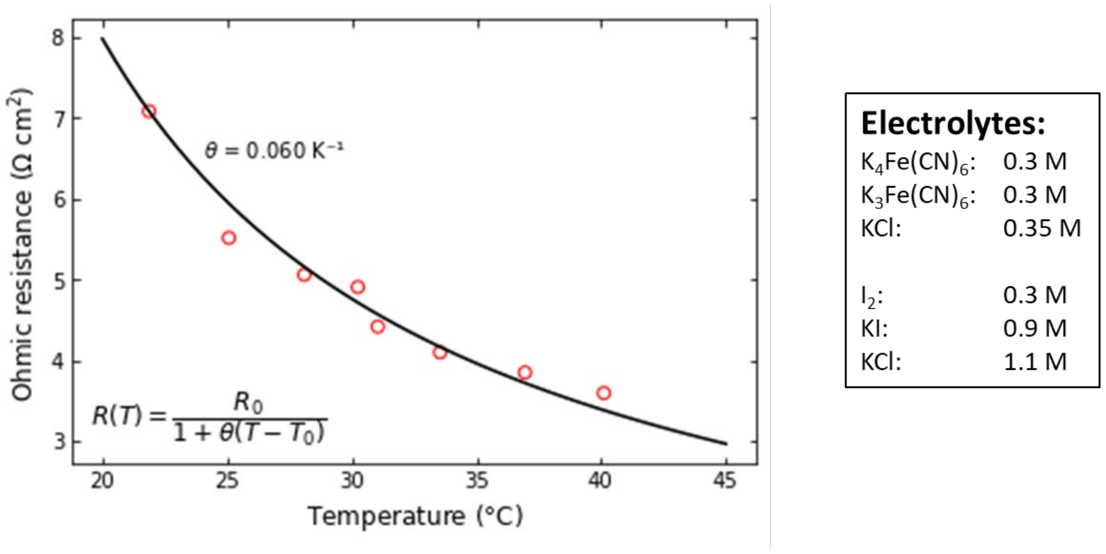

Figure S-3. *Ohmic resistance of a single cell against temperature. The data was using the SciPy curve_fit in Python fitted to find parameter ϑ.*

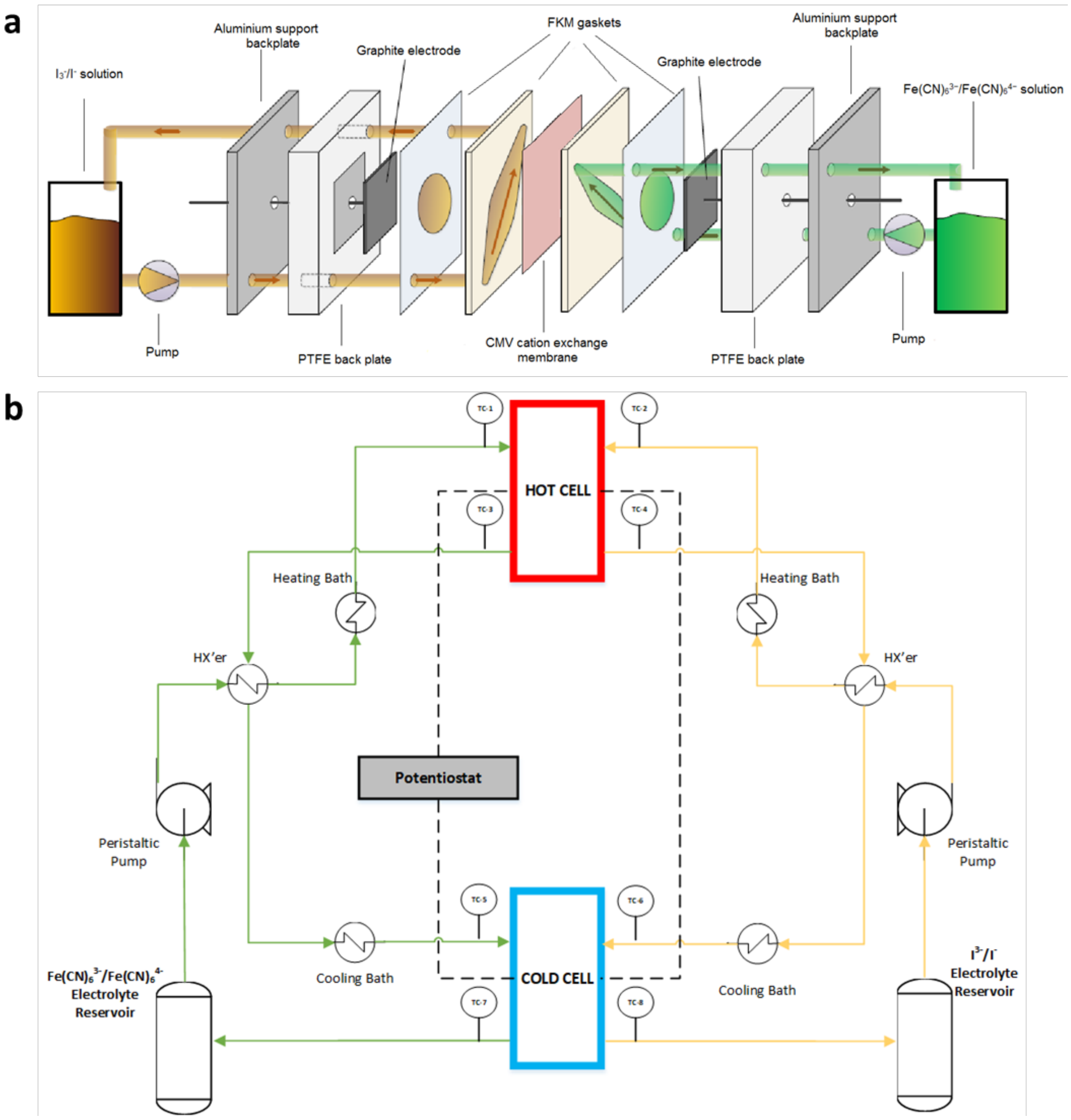

Figure S-4. a. *Exploded view of the used flow cell setup.* ***b.*** *Schematic representation of the two flow cell proof of concept setup, which was used to convert heat into electricity.*

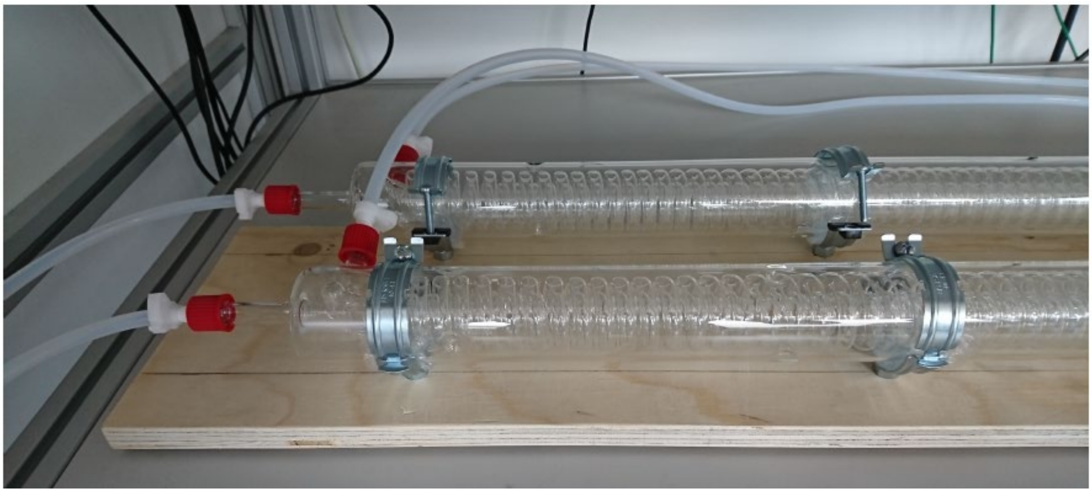

Figure S-5. *Photograph of the glass heat exchangers*

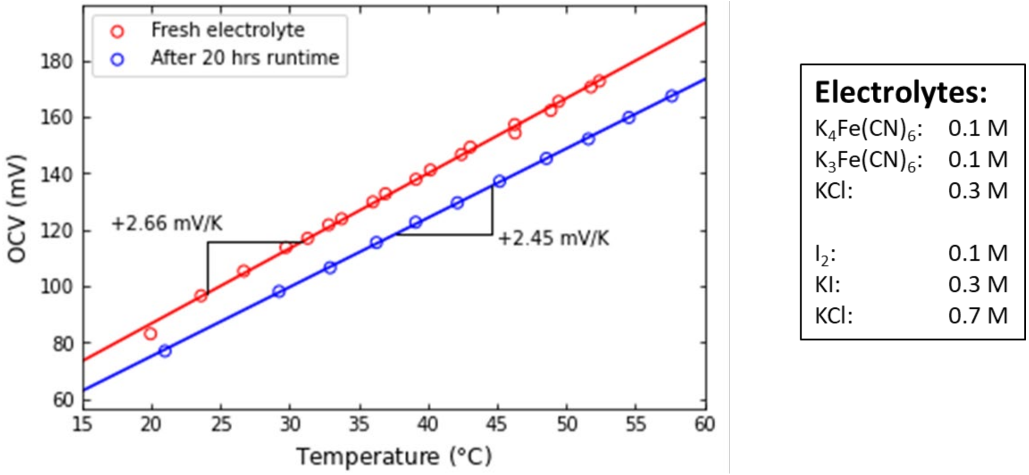

Figure S-6*. Temperature coefficient measured in fresh electrolyte and after 20 hours of measuring.*

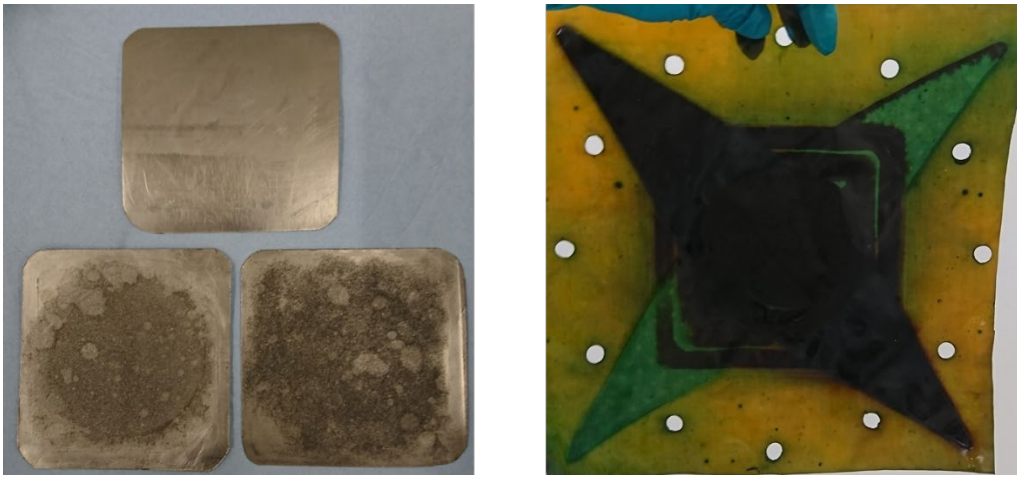

Figure S-7*. Photos of the corrosion on the graphite electrodes (left) and the membrane (right)*

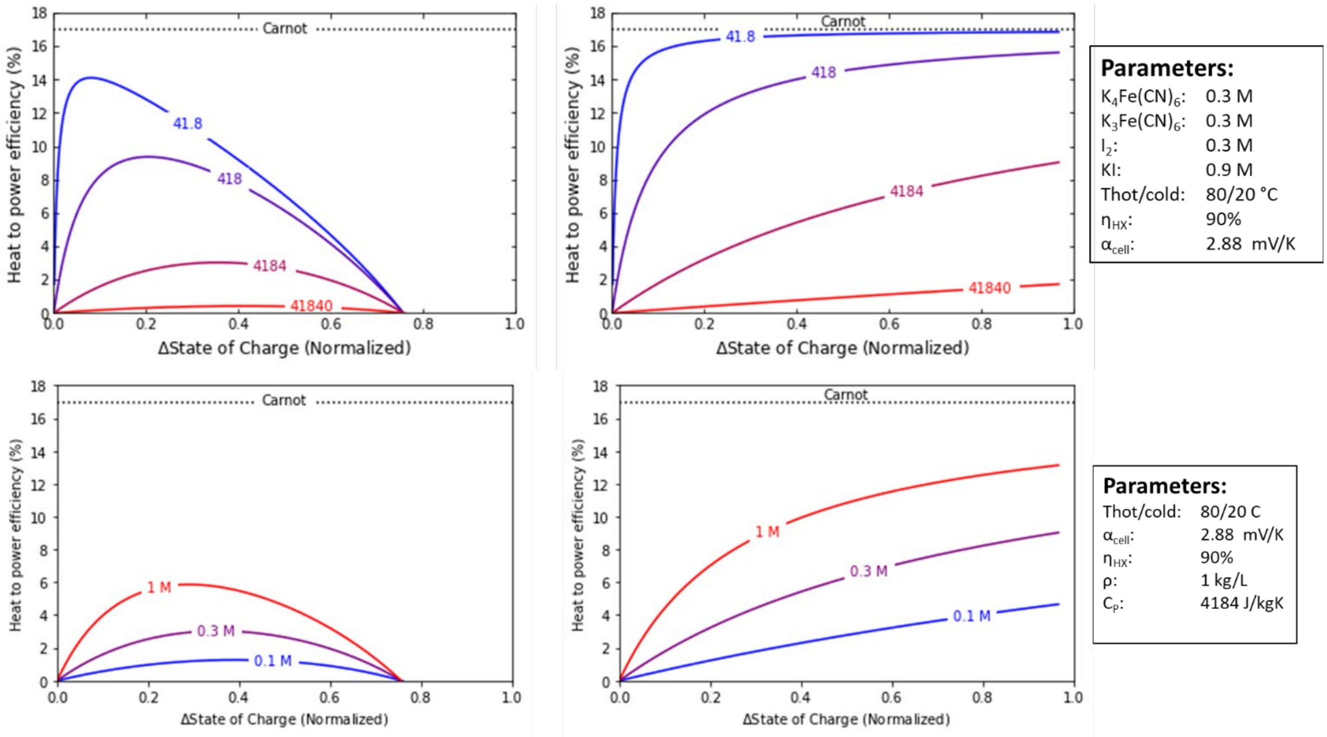

Figure S-8. *Calculated effects of heat capacity (Top) and electrolyte concentration (bottom) on the heat to power efficiency. The left graphs are for a system operating in continuous mode, while the right graphs are for batch mode operation.*

Note: Concentrations are written as per $Fe(CN)_6$ (i.e. 0.1 M = 0.1 M for $Fe(CN)_6^{3-/4-}$, I_2 and 0.3 M for KI)

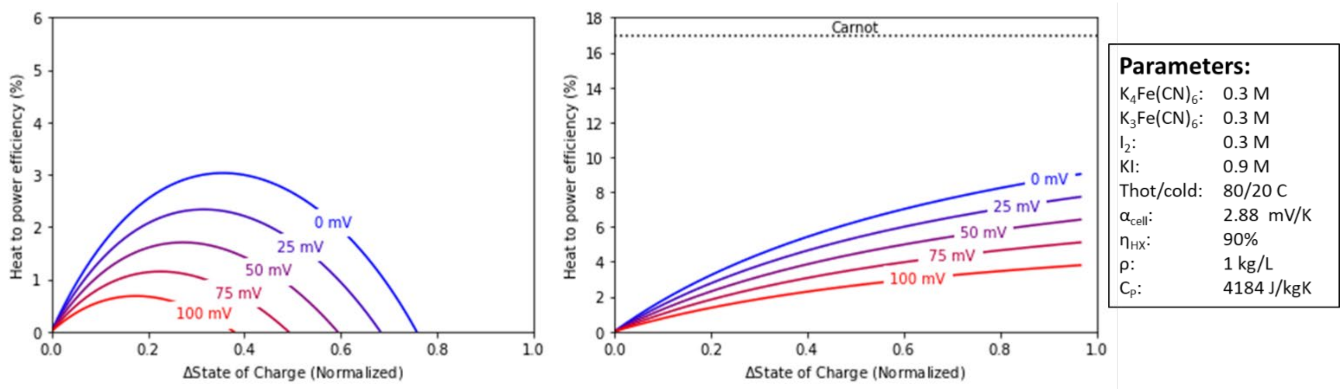

Figure S-9. *Calculated effects of ohmic losses on the heat to power efficiency for a system operating in continuous mode (left) and batch mode (right).*

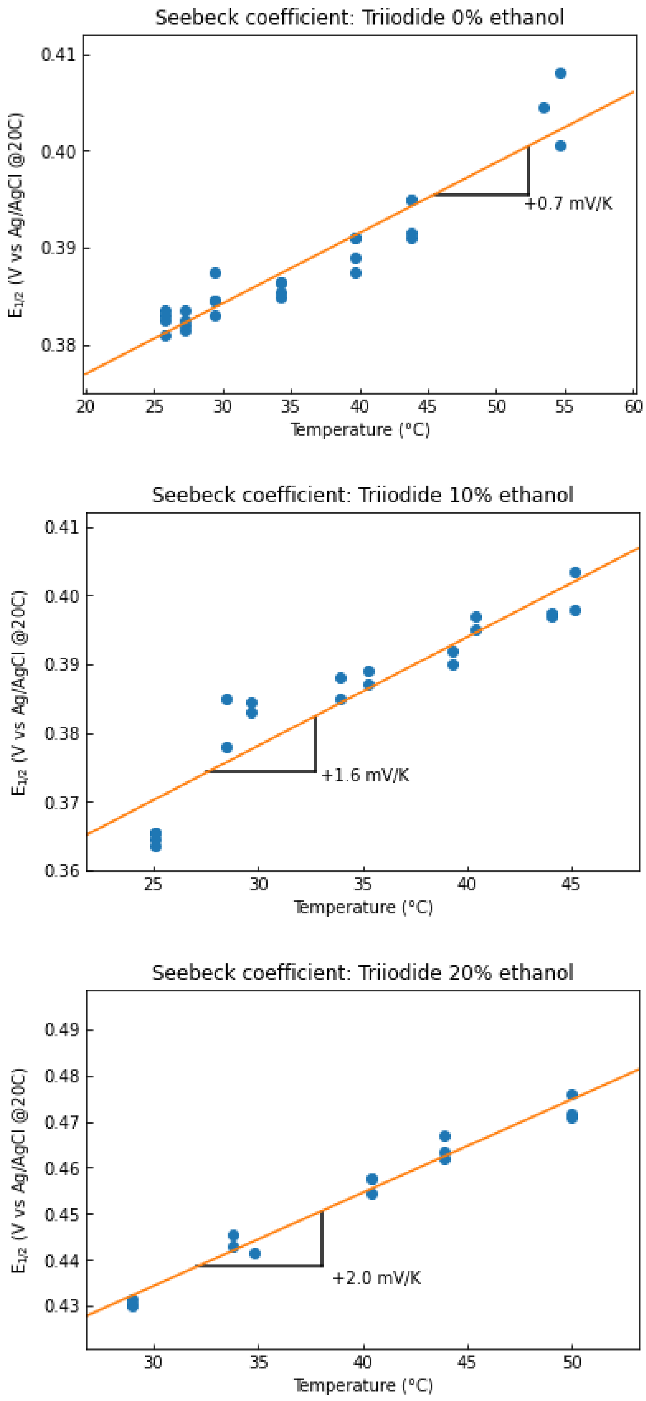

Figure S-10. *Effect of adding a volume fraction of ethanol to the temperature coefficient of the I^-/I_3^- redox couple. The used electrolytes were:*

Top. *1M KI, 0.1 M I_2, 0% EtOH*

Middle. *1M KI, 0.1 M I_2, 10% EtOH*

Bottom. *1M KI, 0.1 M I_2, 20% EtOH*

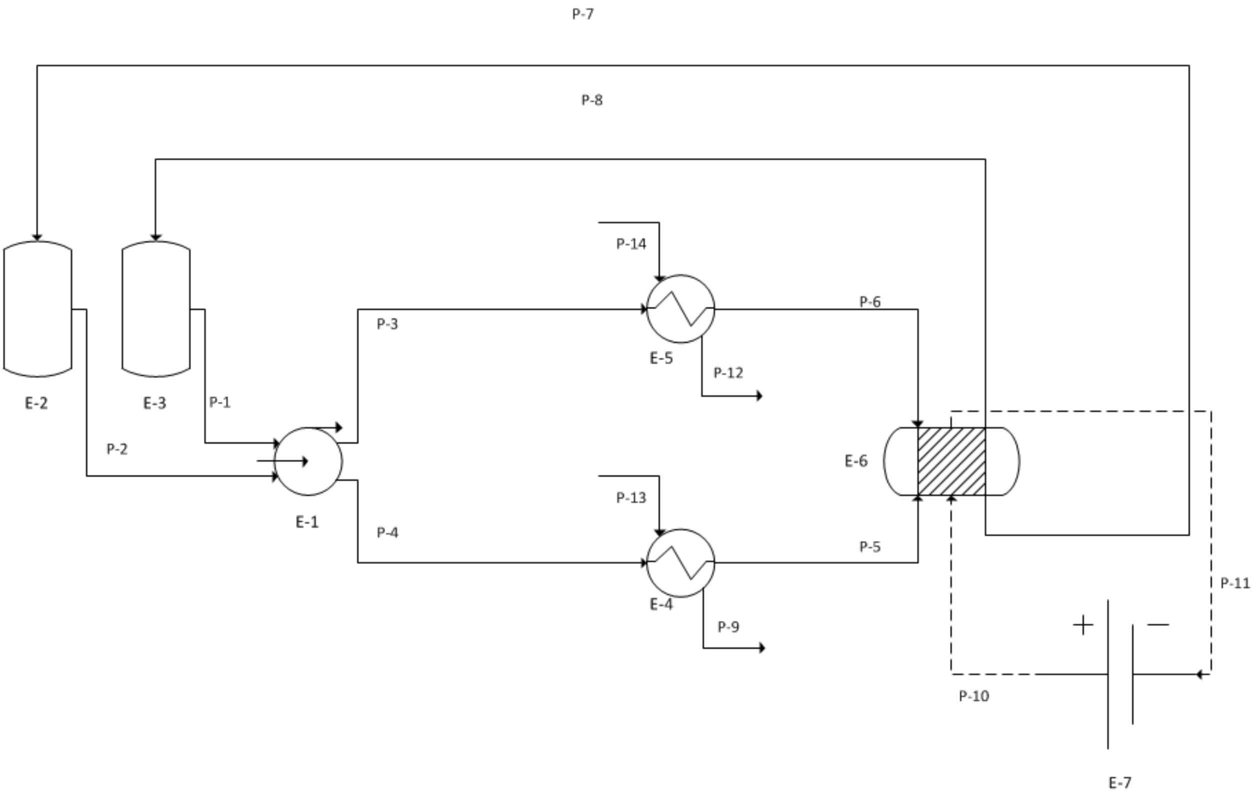

Figure S-11. *Process flow diagram of the single cell experimental setup.*

Equipment number (E-x)	Equipment
**1**	Peristaltic pump
**2**	Electrolyte container (0.5 L)
**3**	Electrolyte container (0.5 L)
**4**	Heating bath
**5**	Heating bath
**6**	Flow cell
**7**	Potentiostat

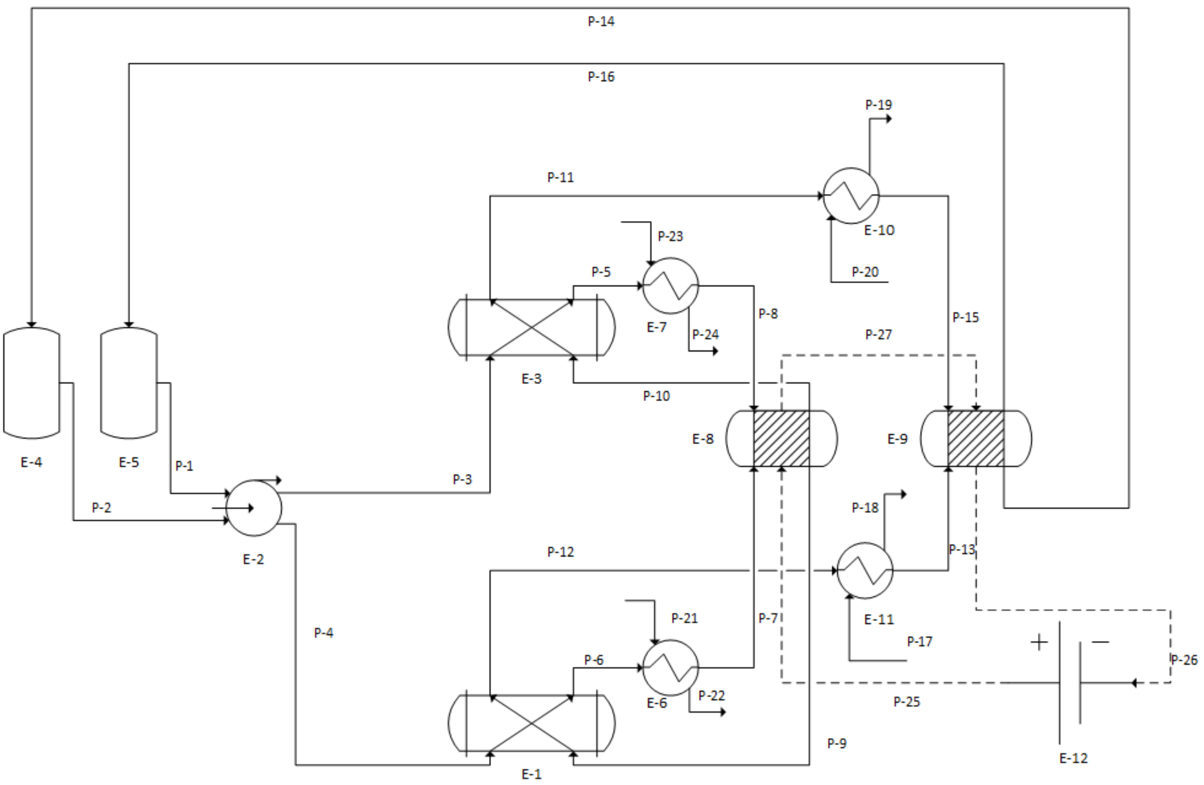

Figure S-12. *Process flow diagram of the two cell experimental setup.*

Equipment number (E-x)	Equipment
**1**	Heat exchanger
**2**	Peristaltic pump
**3**	Heat exchanger
**4**	Electrolyte container (2 L)
**5**	Electrolyte container (2 L)
**6**	Heating bath
**7**	Heating bath
**8**	Hot flow cell
**9**	Cold flow cell
**10**	Cooling bath
**11**	Cooling bath
**12**	Potentiostat

Supplementary Note 1. Temperature coefficient derivation

To derive the equation for the temperature coefficient, we start at the relation between Gibbs energy and the electrochemical potential:

$$E = -\frac{\Delta G}{nF}$$

Here E is the electrochemical potential in V, ΔG is the change in Gibbs energy during the reaction in J/mol, n is the amount of electrons transferred in the reaction and F is Faradays constant.

We then substitute the Gibbs energy with the enthalpy and the entropy:

$$E = T\Delta S - \Delta H$$

Here T is the temperature in K, ΔS is the change in entropy during the reaction in J/(mol K) and ΔH is the change in enthalpy during the reaction in J/mol. The temperature coefficient is the temperature derivative of the electrochemical potential:

$$\alpha = \left(\frac{dE}{dT}\right) = \frac{\Delta S}{nF} + \left(\frac{T\left(\frac{d\Delta S}{dT}\right) - \left(\frac{d\Delta H}{dT}\right)}{nF}\right)$$

Here α is the temperature coefficient of the redox reaction in V/K. Note that Δ represents the change due to reaction and *d* is used for a derivative, e.g. (dΔS/dT) is the first derivative of the reaction entropy to the temperature.

When assuming a constant pressure (dP = 0), the following relations can be used:

$$\left(\frac{\partial \Delta S}{\partial T}\right)_P = \frac{\Delta C_P}{T}$$

$$\left(\frac{d\Delta H}{dT}\right) = d\Delta S = \left(\frac{\partial \Delta S}{\partial T}\right)_P dT + \left(\frac{\partial \Delta S}{\partial P}\right)_T dP = \int \frac{\Delta C_P}{T} dT$$

Here, ΔC_P is the reaction heat capacity in J/(mol K). This results in:

$$\alpha = \frac{\Delta S}{nF} + \frac{\Delta C_P - \int \frac{\Delta C_P}{T} dT}{nF}$$

As noted by Hammond and Risen, the ΔC_P terms in the equation are of similar magnitude and of opposite signs, and tend to have a small effect on the temperature coefficient (up to 8% of ΔS)[1]. Similarly Salvi and deBethune reported a small effect of the reaction heat capacity on the electrode temperature coefficient[2], which allows us to approximate the temperature coefficient to be independent of temperature and mostly dependent on the reaction entropy:

$$\alpha \approx \frac{\Delta S}{nF}$$

To find the concentration dependence of the temperature coefficient we use the Nerst equation, where E_0 is the potential at standard conditions, α_0 the temperature coefficient at standard concentrations and Q the reaction quotient of the redox reactions:

$$E = E_0 - \frac{RT}{nF}\ln(Q)$$

$$\alpha = \frac{dE}{dT} = \alpha_0 - \frac{R}{nF}\ln(Q)$$

When using a calibration value for α_0 we can predict the values for non-standard concentrations. E.g. for the Iodide/Tri-iodide redox couple we can use 0.72 mV/K as α_0, the temperature coefficient for I^-/I_3^- reported in Bratsch (-0.186 mV/K) corrected with the absolute temperature coefficient of the

SHE (+0.906 mV/K). [3] The above equation can be used to predict the temperature coefficients at non-standard concentrations from our work and two literature sources:

Table S-1. *Comparision of reported temperature coefficients of the I^-/I_3^- redox couple*

Source	*[I-] (mol/L)*	*[I3-] (mol/L)*	*ln(Q)*	*α – reported (mV/K)*	*α – calculated* (mV/K)*
Ding et al[4]	*1.9*	*0.1*	*4.23*	*0.53*	*0.54*
Qian et al[5]	*0.9*	*0.1*	*1.99*	*0.63***	*0.63*
This work	*0.009*	*0.001*	*-7.22*	*1.04*	*1.03*

** α is calculated according to $\alpha = \alpha_0 - R/nF \cdot \ln([I^-]^3/[I_3^-])$, where α_0 is 0.72 mV/K and $R/nF = 4.31 \cdot 10^{-2}$ mV/K*

*** This value is predicted, based on the 0.1 mV/K larger cell temperature coefficient of Qian et al*

Supplementary Note 2. Carnot efficiency from TS-diagram

The TS-diagram (Figure 1B of main article) illustrates the work via the area enclosed by the cycle. Step 1 and 3 of this cycle represent the heating and cooling, respectively, in the heat exchanger. A complete heat exchange is assumed (i.e., 100% heat recuperation). As the heat capacity of the cold and hot fluids can be assumed the same (see previous section), this implies that the ΔT for step 1 and 3 are equal. When the external waste heat is added to the hot battery itself, and removed from the cold battery, the discharging at step 2 and charging at step 4 are being performed isothermal. Hence, this cycle corresponds to a trapezium, depicted in Fig. 2B of the main paper.

The area of this trapezium can be calculated via $area = T_H \Delta S_H - T_L \Delta S_L$, where the subscripts H and L indicate High and Low temperature, respectively. As the reaction enthalpy is assumed to be independent of temperature (i.e., $\Delta H_H = \Delta H_L$), which is again justified from the small effect of heat capacity compared to reaction entropy, this area corresponds to the change in Gibbs energy during discharging and charging, i.e., the maximum obtainable work.

Similarly, the heat input, required for step 2, is equal to $T_H \Delta S_H$. Combining these two values yields the heat to power energy efficiency:

$$\eta = \frac{T_H \Delta S_H - T_L \Delta S_L}{T_H \Delta S_H}$$

Because the reaction entropy during discharging and charging can also be assumed the same (i.e.,), this simplifies to

$$\eta = \frac{T_H - T_L}{T_H} = 1 - \frac{T_L}{T_H}$$

Which is equal to the Carnot efficiency. Because this derivation has assumed a 100% heat recuperation and assumed that the work is equal to the Gibbs energy change, this corresponds to an idealized case without Ohmic losses and heat losses.

Supplementary Note 3. Temperature coefficients for simple redox reactions

We collected temperature coefficient data from literature, to investigate our theory shown in Figure 2C, which says:

- Simple redox couples consisting of cations have a positive temperature coefficient
- Simple redox couples consisting of anions have a negative temperature coefficient

As of now, we did not find any exceptions to the theory, and it seems that for most simple redox reactions the entropy change is mainly caused by a change in hydration shell. See figure S-13and table S-2:

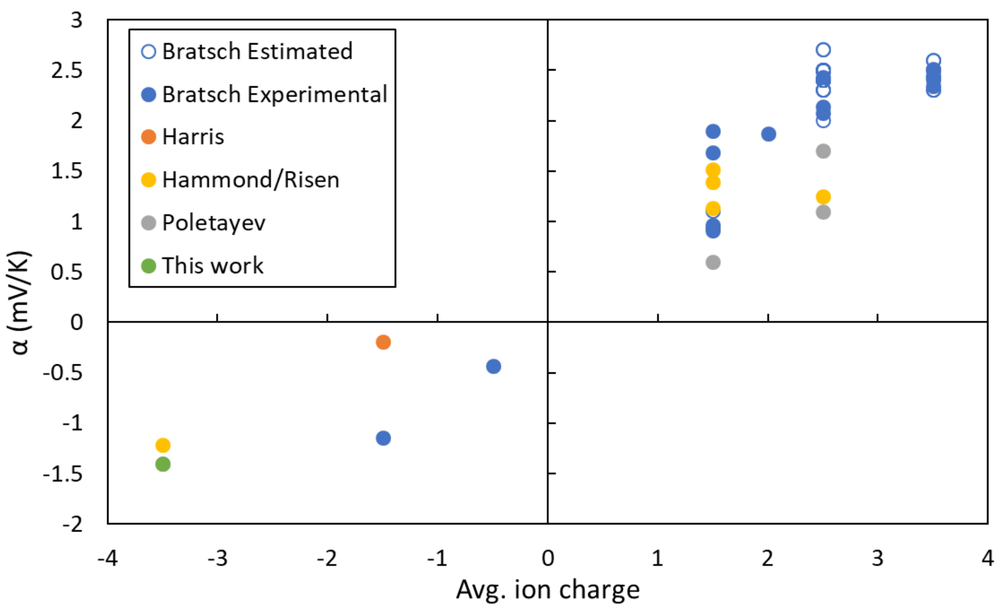

Figure S-13. Comparision of temperature coefficient with the average charge of the redox-active species of a redox reaction (e.g. average charge is +2.5 for Fe^{3+}+ e^- → Fe^{2+}). The data this figure contains estimated and experimental temperature coefficients from Bratsch [3], and experimental values from Harris[6], Hammond & Risen[1] and this work.

Table S-2. *Tabulated temperature coefficients from Bratsch[3], Harris[6], Hammond/Risen[1], Poletayev[7] and this work.*

Redox couple	*$(dV/dT)_{isothermal}$** *mV / K*	*α* *mV / K*	*Avg Charge*	*Source*	*Data type*
Pr4+ / Pr3+	+1.4	+2.3	+3.5	Bratsch	Estimated
Tb4+ / Tb3+	+1.5	+2.4	+3.5	Bratsch	Estimated
Pa4+ / Pa3+	+1.7	+2.6	+3.5	Bratsch	Estimated
Am4+ / Am3+	+1.4	+2.3	+3.5	Bratsch	Estimated
Cm4+ / Cm3+	+1.7	+2.6	+3.5	Bratsch	Estimated
Bk4+ / Bk3+	+1.6	+2.5	+3.5	Bratsch	Estimated
Cf4+ / Cf3+	+1.6	+2.5	+3.5	Bratsch	Estimated
Sc3+ / Sc2+	+1.6	+2.5	+2.5	Bratsch	Estimated
Pr3+ / Pr2+	+1.6	+2.5	+2.5	Bratsch	Estimated
Nd3+ / Nd2+	+1.6	+2.5	+2.5	Bratsch	Estimated
Pm3+ / Pm2+	+1.5	+2.4	+2.5	Bratsch	Estimated
Sm3+ / Sm2+	+1.4	+2.3	+2.5	Bratsch	Estimated
Dy3+ / Dy2+	+1.6	+2.5	+2.5	Bratsch	Estimated

Ho3+ / Ho2+	+1.6	+2.5	+2.5	Bratsch	Estimated
Er3+ / Er2+	+1.6	+2.5	+2.5	Bratsch	Estimated
Tm3+ / Tm2+	+1.6	+2.5	+2.5	Bratsch	Estimated
Yb3+ / Yb2+	+1.4	+2.3	+2.5	Bratsch	Estimated
Pu3+ / Pu2+	+1.5	+2.4	+2.5	Bratsch	Estimated
Am3+ / Am2+	+1.8	+2.7	+2.5	Bratsch	Estimated
Bk3+ / Bk2+	+1.6	+2.5	+2.5	Bratsch	Estimated
Cf3+ / Cf2+	+1.6	+2.5	+2.5	Bratsch	Estimated
Es3+ / Es2+	+1.6	+2.5	+2.5	Bratsch	Estimated
Fm3+ / Fm2+	+1.6	+2.5	+2.5	Bratsch	Estimated
Md3+ / Md2+	+1.6	+2.5	+2.5	Bratsch	Estimated
No3+ / No2+	+1.5	+2.4	+2.5	Bratsch	Estimated
Ti3+ / Ti2+	+1.5	+2.4	+2.5	Bratsch	Estimated
V3+ / V2+	+1.5	+2.4	+2.5	Bratsch	Estimated
Cr3+ / Cr2+	+1.4	+2.3	+2.5	Bratsch	Estimated
Mn3+ / Mn2+	+1.8	+2.7	+2.5	Bratsch	Estimated
Ni3+ / Ni2+	+1.1	+2.0	+2.5	Bratsch	Estimated
Cu3+ / Cu2+	+1.5	+2.4	+2.5	Bratsch	Estimated
(UO2)2+ / (UO2)+	+0.2	+1.1	+1.5	Bratsch	Estimated
Ce4+ / Ce3+	+1.54	+2.45	+3.5	Bratsch	Experimental
U4+ / U3+	+1.61	+2.52	+3.5	Bratsch	Experimental
Np4+ / Np3+	+1.53	+2.44	+3.5	Bratsch	Experimental
Pu4+ / Pu3+	+1.441	+2.347	+3.5	Bratsch	Experimental
Eu3+ / Eu2+	+1.53	+2.44	+2.5	Bratsch	Experimental
Fe3+ / Fe2+	+1.175	+2.081	+2.5	Bratsch	Experimental
Co3+ / Co2+	+1.23	+2.14	+2.5	Bratsch	Experimental
Tl3+ / Tl+	+0.97	+1.90	+2	Bratsch	Experimental
(NpO2)2+ / (NpO2)+	+0.058	+0.964	+1.5	Bratsch	Experimental
(PuO2)2+ / (PuO2)+	+0.03	+0.94	+1.5	Bratsch	Experimental
(AmO2)2+ / (AmO2)+	+0.01	+0.92	+1.5	Bratsch	Experimental
Cu2+ / Cu+	+0.776	+1.682	+1.5	Bratsch	Experimental
Ag2+ / Ag+	+0.99	+1.90	+1.5	Bratsch	Experimental
(ClO2) / (ClO2)-	-1.335	-0.429	-0.5	Bratsch	Experimental
(MnO4)- / (MnO4)2-	-2.05	-1.14	-1.5	Bratsch	Experimental
Fe3+ / Fe2+ (Salicylamide)	*N/A*	+1.25**	+2.5	Hammond/Risen	Experimental
Cu2+ / Cu+ (Acetate)	*N/A*	+1.13**	+1.5	Hammond/Risen	Experimental
Cu2+ / Cu+ (Pyridine)	*N/A*	+1.51**	+1.5	Hammond/Risen	Experimental
Cu2+ / Cu+ (NH3)	*N/A*	+1.39**	+1.5	Hammond/Risen	Experimental
[Fe(CN)6]3- / [Fe(CN)6]4-	*N/A*	-1.21**	-3.5	Hammond/Risen	Experimental
Mn(EDTA)- / Mn(EDTA)2-	-1.1	-0.19	-1.5	Harris	Experimental
V3+/V2+	*N/A*	+1.7	+2.5	Poletayev	Experimental
Fe3+/Fe2+	*N/A*	+1.1	+2.5	Poletayev	Experimental
Methyl Viologen (2+/1+)	*N/A*	+0.6	+1.5	Poletayev	Experimental
[Fe(CN)6]3- / [Fe(CN)6]4-	*N/A*	-1.4	-3.5	Poletayev	Experimental
[Fe(CN)6]3- / [Fe(CN)6]4-	*N/A*	-1.4	-3.5	This work	Experimental

**Bratsch and Harris reported isothermal temperature coefficients, they have been converted into the temperature coefficient by adding the Seebeck coefficient of the SHE (0.906 mV/K^3)*

***Hammond & Risen reported ΔS values of the redox reactions, these have been converted into the temperature coefficient with equation 2.*

Supplementary Note 4. Figure of merit calculation:

The figure of merit is a dimensionless constant to indicate the relative energy gain to the total heating of the system. Figure of merit (Y) calculations were done as per methods of Lee *et al* [8]:

$$Y = \frac{\alpha q_c}{c_P}$$

Here Y is the figure of merit, α is the total temperature coefficient, q_c is the charge capacity and heat capacity per unit volume. For solid electrodes, it is more convenient to do this calculation per unit of mass.

Figure of merit calculation for our system

$\alpha = |\alpha_{cell}| = 2.88$ mV K^{-1}

Specific charge capacity is calculated for various ΔSOC, as the battery in continuous mode requires a ΔSOC of less than 0.75 to have a positive energy gain. Specific charge capacity is then defined as:

$$q_c = \frac{\Delta SOC \cdot total\ charge}{total\ volume}$$

As ferro/ferri-cyanide is the limiting reactant in our system, it will be used in total charge. For ease of calculation, 1L of both electrolytes is assumed.

1 L of 0.3M $K_3Fe(CN)_6$, 0.3M $K_4Fe(CN)_6$, 0.35M KCl and 1L of 0.3M I_2, 0.9M KI, 1.1M KCl.

$q_c = \Delta SOC \cdot ([Fe(CN)_6^{3-}] + [Fe(CN)_6^{4-}])\cdot n \cdot F/V_{total} = \Delta SOC \cdot (0.3M + 0.3M)\cdot 1\cdot 96485\ C\ mol^{-1}/2\ L =$ **$\Delta SOC \cdot 28945\ C\ L^{-1}$**

The heat capacity of our electrolytes was calculated using standard heat capacities reported by Hepler and Hovey[9]:

- $K_3Fe(CN)_6$ = -215 J mol^{-1} K^{-1} [9]
- $K_4Fe(CN)_6$ = -475 J mol^{-1} K^{-1} [9]
- KCl = -114 J mol^{-1} K^{-1} [9]
- KI = -109 J mol^{-1} K^{-1} [9]
- KI_3 = +170 J mol^{-1} K^{-1} [10] - assumed all I_2 reacted with KI

The total heat capacity is:

$c_P = (c_P(2\ L\ water) + c_P(0.3\ mol\ K_3Fe(CN)_6) + c_P(0.3\ mol\ K_4Fe(CN)_6) + c_P(1.45\ mol\ KCl) + c_P(0.6\ mol\ KI) + c_P(0.3\ mol\ KI_3)/total\ volume\ (2L) = (2\cdot 4184 - 0.3\cdot 215 - 0.3\cdot 475 - 1.45\cdot 114 - 0.6\cdot 109 + 0.3\cdot 170)/2 =$ **$3991\ J\ L^{-1}\ K^{-1}$**

$Y = 2.88 \cdot 10^{-3}\ V\ K^{-1} \cdot \Delta SOC \cdot 28945\ C\ L^{-1} / 3991\ J\ L^{-1}\ K^{-1}$

**ΔSOC**	**Y**
0.25	**0.005**
0.5	**0.010**
1	**0.021**

Similar to the system of for Hammond and Risen [1], the concentration of Fe(CN)6 could be increased to 0.5 M each. Assuming that the temperature coefficient and heat capacity are not affected by that change, this would increase the Y to 0.033 for ΔSOC = 1.

Figure of merit calculation for $Fe(CN)_6^{3-/4-}$ & $Cu(NH_3)_4^{2+}/Cu(NH_3)_2^{+}$ [1]

The volume based Figure of Merit calculation for the system for Hammond and Risen [1] was done similar to our system:

α = 2.9 mV K^{-1}

Hammond and Risen used 0.5 M of $Cu^{1+/2+}$ and 0.5 M of $Fe(CN)_6^{3-/4-}$, so neither compound is limiting reactant, No information was given on the heat capacity, so assumed to be similar to water

- $q_c = ([Fe(CN)_6^{3-}] + [Fe(CN)_6^{4-}])\cdot n\cdot F/V_{total} = (0.5\ M + 0.5\ M)\cdot 1\cdot 96485\ C\ mol^{-1} / 2\ L = 48243\ C\ L^{-1}$
- $c_P = 4184\ J\ K^{-1}\ L^{-1}$

$Y = 2.9 \cdot 10^{-3}\ V\ K^{-1} \cdot 48243\ C\ L^{-1} / 4184\ J\ K^{-1}\ L^{-1} =$ **0.033**

Figure of merit calculation for $Fe(CN)_6^{3-/4-}$, FeHCF [11]

Parameters are taken from the article of Yang *et al* [11]. The heat capacity values were calculated using the supplementary information, assuming a liquid/solid ratio of 1 mL g^{-1} (i.e. 1 g Prussian blue & 1 mL $Fe(CN)_6$ solution). The heat capacity of the solution and Prussian blue are 1.1 J g^{-1} K^{-1} and 4.18 J cm^{-3} K^{-1} respectively. Assuming an electrolyte density of 1 g cm^{-3}, the average heat capacity per gram is (1.1 + 4.18)/2 = 2.64 J g^{-1} K^{-1}

- α = 1.45 mV K^{-1}
- q_c = 30.0 mAh g^{-1} = 108 C g^{-1}
- c_P = 2.64 J g^{-1} K^{-1}

$Y = 1.45 \cdot 10^{-3}\ V\ K^{-1} \cdot 108\ C\ g^{-1} / 2.64\ J\ K^{-1}\ g^{-1} =$ **0.059**

Figure of merit calculation for NiHCF, Ag/AgCl system [12]

Parameters are taken from the article of Yang *et al* [12]:

- α = 0.74 mV K^{-1}
- q_c = 36.0 mAh g^{-1} = 129.6 C g^{-1}
- c_P = 2.84 J g^{-1} K^{-1}

$Y = 0.74 \cdot 10^{-3}\ V\ K^{-1} \cdot 129.6\ C\ g^{-1} / 2.84\ J\ g^{-1}\ K^{-1} =$ **0.034**

Figure of merit calculation for $Fe(CN)_6^{3-/4-}$, V^{3+}/V^{2+} *system* [7]

Parameters are taken from the SI of Poletayev *et al* [7]

- α = 3.00 mV K^{-1}
- $q_c = ([Fe(CN)_6^{3-}] + [Fe(CN)_6^{4-}])\cdot n\cdot F/V_{total} = 0.8\ mol\ L^{-1}\cdot 1\cdot 96485\ C\ mol^{-1}/2\ L = 38594\ C\ L^{-1}$
- c_P = 3.0 J g^{-1} K^{-1}
- $\rho_{avg} = (1160 + 1290\ g\ L^{-1})/2 = 1225\ g\ L^{-1}$

$Y = 3.00 \cdot 10^{-3}\ V\ K^{-1} \cdot 38594\ C\ L^{-1} / 1225\ g\ L^{-1} / 3.0\ J\ g^{-1}\ K^{-1} =$ **0.032**

Figure of merit calculation for VO_2^+/VO^{2+}, V^{3+}/V^{2+} *system* [13]

Parameters are taken from the paper of Reynard *et al*[13]*:*

- α = 1.16 mV K^{-1}
- c_P = 4200 J L^{-1} K^{-1}
- q_c = 1 M total Vanadium·n·F/V_{total} = 1 mol L^{-1}·1·96485 C mol^{-1}/2 L = 48243 C L^{-1}

Y = 1.16 · 10^{-3} V K^{-1} · 48242 C L^{-1} / 4200 J L^{-1} K^{-1} = **0.013**

Figure of merit calculation for the $Fe(CN)_6^{3-/4-}$ & I^-/I_3^- *system of Qian et al*[5]

Parameters are taken from Qian *et al*[5]

No heat capacity was reported, assumed similar to water:

- c_P = 4184 J K^{-1} L^{-1}
- α = 1.9 mV K^{-1}
- q_c = ($[Fe(CN)_6^{3-}]$ + $[Fe(CN)_6^{4-}]$)·n·F/V_{total} = 0.75 mol L^{-1}·1·96485 C mol^{-1}/2 L = 36182 C L^{-1}

Y = 1.9 · 10^{-3} V K^{-1} · 36182 C L^{-1} / 4184 J L^{-1} K^{-1} = **0.016**

Supplementary Note 5. Derivation of maximum Power and energy losses

To derive the maximum power we define the cell voltage in three parts:

- The open cell voltage , E_{OC}, which is the cell voltage at zero current
  - For our cell $E_{OC} = \alpha\Delta T$
- The current dependent losses:
  - The Ohmic losses, IR, which is the current times the Ohmic resistance
  - The charge transfer losses, $\eta_{CT}(I)$, in the form of a reaction overpotential
- The current independent losses:
  - The concentration overpotential, RT/nF ln(Q).

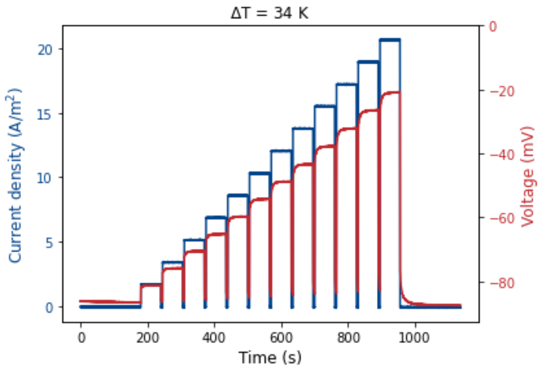

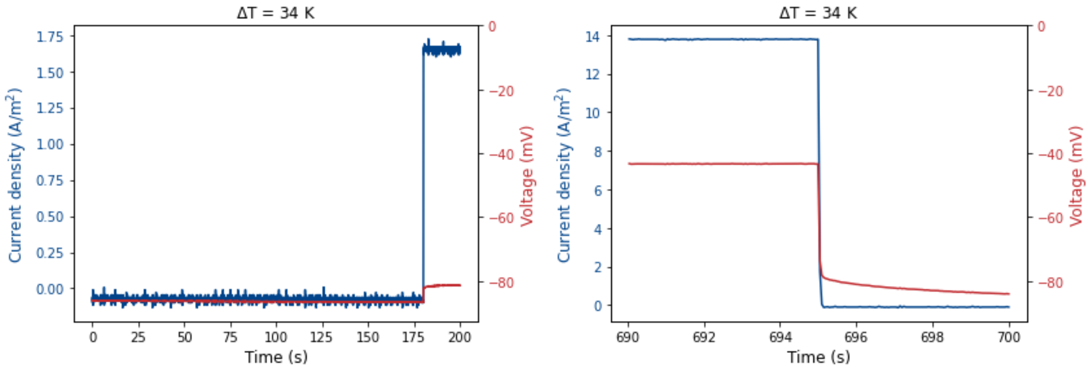

Figure S-13. Current/Voltage time curves for the system. Cold and hot cell operate at 20 and 54 °C respectively

For a discharging battery, the voltage then becomes:

$$U = E_{OC} - IR - \eta_{CT}(I) + \frac{RT}{nF}\ln(Q) \tag{1}$$

We can measure E_{OC} at the start of the measurements where the concentrations are still unchanged. When interrupting the current, the IR and $\eta_{CT}(I)$ terms become 0, and what is left is the E_{OC} and the RT/nF lnQ term.

At the maximum power density the temperature and current density are equal to ΔT = 34 K and j = 13.8 A/m^2.

The OCV at 0 A/m^2 is 86.6 mV, U at 13.8 A/m^2 is 43.4 mV, and U after the current interrupt is 78.2 mV.

The current dependent voltage change is 35 mV and the current independent change is 8.4 mV. This means that 10% of energy is lost due to concentration overpotential, and 40% due to Ohmic and activation overpotential losses. At maximum power density, the temperature of the hot and cold cell are 20 °C and 54 °C respectively, which results in a Carnot efficiency of 10.4%. As half of the energy is lost, the maximum heat to power efficiency at this power density is 5.2%.

Supplementary Note 6. Explanation of the Python model:

The calculations for Figure 4 a & b were done in two steps:

1. The calculation of the cell potential at 20 and 80 °C for all state of charge, for a certain starting concentration of redox active species.
2. Calculation of the heat to power efficiency for the full range of Δstate of charge and heat exchanger efficiencies

1. Calculation of the cell potential

The cell potential was calculated with Nernst equation modified with a temperature coefficient. For a general half reaction, a·A(aq) + n·e^- → b·B(aq) where unity activity coefficients are assumed, this equals:

$$E_{\frac{1}{2}}(T) = E_{\frac{1}{2}}^{0} + (T - T^{0}) \cdot \alpha + \frac{RT}{nF}\ln\left(\frac{[A]^{a}}{[B]^{b}}\right)$$

- $E_{1/2}(T)$ is the half-reaction potential at temperature T and concentrations [A] & [B]
- $E_{1/2}^0$ is the standard half-reaction potential at 25 °C (298.15 K) and 1 M concentrations
- R, Ideal gas constant (8.314 J $mol^{-1}K^{-1}$)
- T, Temperature in degrees Kelvin (T_0 = 298.15 K)
- α, temperature coefficient of half reaction in V K^{-1}
- n, number of electrons that are transferred in the half reaction
- F, Faraday constant – 96485 C mol^{-1}
- [A]&[B], concentration of A and B in mol L^{-1}
- a & b, stoichiometry of A & B in the half reaction

The $E_{1/2}^0$ of the ferrocyanide and triiodide reactions are as follows:

- I_3^- + $2e^-$ → 3 I^- E^0 = 0.54 V vs NHE
- $Fe(CN)_6^{3-}$ + e^- → $Fe(CN)_6^{4-}$ E^0 = 0.36 V vs NHE

When writing down the cell reaction for a spontaneous (galvanic) reaction, for n = 1, you get:

$$0.5I_3^- + Fe(CN)_6^{4-} \rightarrow 1.5I^- + Fe(CN)_6^{3-}$$

The cell potential then becomes:

$$E_{cell}(T) = E_{I_3} - E_{Fe(CN)_6} = E_{cell}^{0} + (T - T^{0}) \cdot \alpha_{cell} + \frac{RT}{nF}\ln\left(\frac{[I_3^-]^{0.5}[Fe(CN)_6^{4-}]}{[I^-]^{1.5}[Fe(CN)_6^{3-}]}\right)$$

- n = 1
- E_{cell}^0, cell potential (0.54 – 0.36 V = 0.18 V)
- α_{cell}, +2.88 mV/K, as measured in figure 2B for the below concentrations
  - $K_4Fe(CN)_6$ 0.3 M
  - $K_3Fe(CN)_6$ 0.3 M
  - I_2 0.3 M
  - KI 0.9 M (results in 0.6 M I^- + 0.3 M I_3^-)

Using the $E_{cell}(T)$ formula, we created the following V-dQ graph:

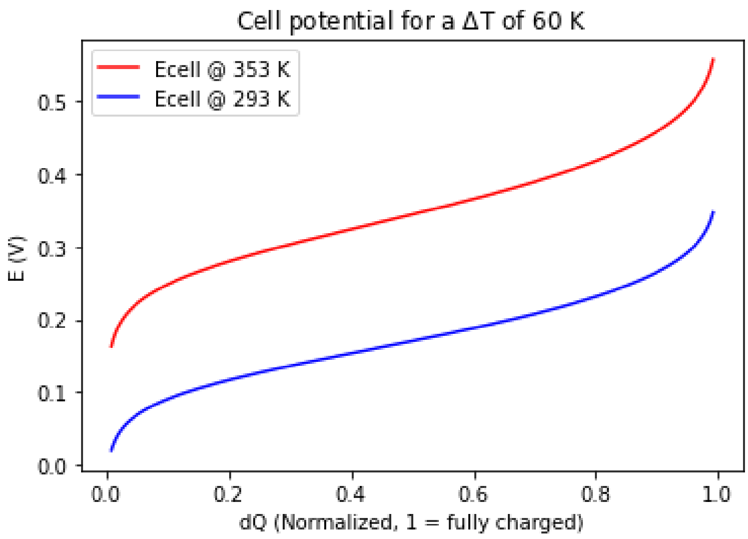

Here dQ is the normalized state of charge, which can be converted into the concentrations (mol L^{-1}) using the table below:

dQ	$[Fe(CN)_6^{4-}]$	$[Fe(CN)_6^{3-}]$	$[I_3^-]$	$[I^-]$
**0**	0	0.6	0.15	1.05
**0.25**	0.15	0.45	0.225	0.825
**0.50**	0.3	0.3	0.3	0.6
**0.75**	0.45	0.15	0.375	0.425
**1**	0.6	0	0.45	0.15

2. Calculation of the heat to power efficiency

For a continuous system, without segmented electrodes, the entire flow cell will operate at the voltage of the flow cell outlet. This can be illustrated with the below graph, where E_1 and E_2 indicate operating voltages of the hot and cold flow cell respectively:

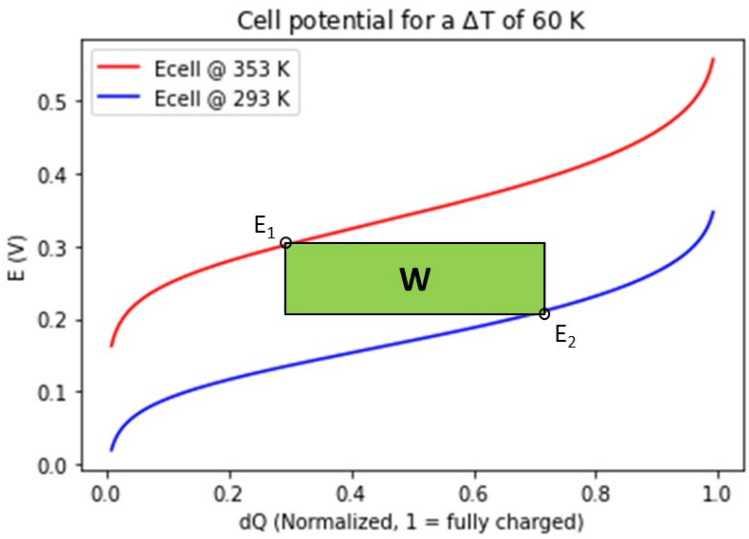

In a batch system, the full surface of the V-dQ can be harvested, which will result in a figure like **Figure 1c**.

The energy costs of heating and cooling have a set value, and the energy gain per cycle is strongly dependent on the extend of (dis)charge (ΔSOC). For the sake of the model we chose that the ΔSOC would be around a normalized dQ of 0.5, which results in the following table:

ΔSOC	dQ-charged	dQ-discharged
**0**	0.5	0.5
**0.2**	0.6	0.4
**0.5**	0.75	0.25
**0.8**	0.90	0.10
**1**	1	0

The efficiency is calculated as:

$$\eta = \frac{W}{Q_{in\ HX} + Q_{in\ reaction}}$$

For a system operating in **continuous mode**, Q_{in} and W are as follows:

$$W = (E_1 - E_2) \cdot \Delta n \cdot n \cdot F$$

$$Q_{in\ HX} = \rho \cdot C_p \cdot V \cdot \Delta T \cdot (1 - \eta_{HX})$$

$$Q_{in\ reaction} = \Delta S \cdot T_H \cdot \Delta n$$

For a system operating in **batch mode**, W changes to:

$$W = \Delta S \cdot \Delta T \cdot \Delta n$$

We chose a total volume of 2 L electrolyte (1 L of each electrolyte), so the concentrations are equal to the amount of moles. The symbols in the above equations:

- W — Generated energy by the TREC in Joules
- $Q_{in\ HX}$ — Extra energy required to heat up system to operating temperature after imperfect heat exchanger, in Joules
- $Q_{in\ reaction}$ — Required energy for endothermic reaction in hot flow cell, in J
- E_1&E_2 — Operating voltage of the hot & cold flow cell as per the figure above, in Volts
- Δn — Amount of moles that react at this ΔSOC, in mol
- n — Number of electrons that are transferred in the half reaction
- F — Faraday constant, 96485 C mol^{-1}
- ρ — Density, 1 kg L^{-1}
- C_P — Heat capacity, 4184 J kg^{-1} K^{-1}
- V — Electrolyte volume, 2 L
- ΔT — Temperature difference between hot and cold flow cells, in degrees Kelvin
- η_{HX} — Efficiency of the heat exchanger
- ΔS — Entropy change of the reaction, in J mol^{-1} K^{-1}
- T_H — Temperature of the hot cell

All these calculations where implemented into a python script, which resulted in the figures in the paper and supplementary information.

References

1. Hammond RH, Risen WM. An electrochemical heat engine for direct solar energy conversion. *Solar Energy* 1979, **23**(5)**:** 443-449.

2. Salvi GR, deBethune AJ. The Temperature Coefficients of Electrode Potentials. *Journal of The Electrochemical Society* 1961, **108**(7)**:** 672.

3. Bratsch S. Standard Electrode Potentials and Temperature Coefficients in Water at 298.15 K. *Journal of Physical and Chemical Reference Data* 1989, **18:** 1-21.

4. Ding Y, Guo X, Ramirez-Meyers K, Zhou Y, Zhang L, Zhao F*, et al.* Simultaneous energy harvesting and storage via solar-driven regenerative electrochemical cycles. *Energy & Environmental Science* 2019, **12**(11)**:** 3370-3379.

5. Qian X, Shin J, Tu Y, Zhang JH, Chen G. Thermally Regenerative Electrochemically Cycled Flow Batteries with pH Neutral Electrolytes as for Harvesting Low-Grade Heat. *Physical Chemistry Chemical Physics* 2021.

6. Harris DC. *Quantitative chemical analysis*. Freeman: New York, 2003.

7. Poletayev AD, McKay IS, Chueh WC, Majumdar A. Continuous electrochemical heat engines. *Energy & Environmental Science* 2018, **11**(10)**:** 2964-2971.

8. Lee SW, Yang Y, Lee H-W, Ghasemi H, Kraemer D, Chen G*, et al.* An electrochemical system for efficiently harvesting low-grade heat energy. *Nature Communications* 2014, **5**(1)**:** 3942.

9. Hepler L, Hovey J. Standard state heat capacities of aqueous electrolytes and some related undissociated species. *Canadian Journal of Chemistry* 2011, **74:** 639-649.

10. Lemire RJ, Campbell AB, Saluja PPS, LeBlanc JC. Heat capacities and densities of electrolyte mixtures in aqueous solution — Application to the determination of apparent molar heat capacities and volumes for potassium triiodide and dioxoneptunium(V) perchlorate. *Journal of Nuclear Materials* 1993, **201:** 165-175.

11. Yang Y, Lee SW, Ghasemi H, Loomis J, Li X, Kraemer D*, et al.* Charging-free electrochemical system for harvesting low-grade thermal energy. *Proc Natl Acad Sci U S A* 2014, **111**(48)**:** 17011-17016.

12. Yang Y, Loomis J, Ghasemi H, Lee SW, Wang YJ, Cui Y*, et al.* Membrane-Free Battery for Harvesting Low-Grade Thermal Energy. *Nano Letters* 2014, **14**(11)**:** 6578-6583.

13. Reynard D, Dennison CR, Battistel A, Girault HH. Efficiency improvement of an all-vanadium redox flow battery by harvesting low-grade heat. *Journal of Power Sources* 2018, **390:** 30-37.
